# Oral delivery of recombinant *Lactococcus lactis* expressing an IL-23 inhibitor as a potential therapeutic strategy for experimental colitis

**DOI:** 10.1080/10717544.2026.2726654

**Published:** 2026-10-03

**Authors:** Eliska Kopecna, Leona Raskova Kafkova, Petr Kosztyu, Kristyna Sloupenska, Katerina Zachova, Alica Cutkova, Jozef Skarda, Jana Vaculova, Milan Kuchar, Natalya Panova, Stephen Walimbwa, Tina Vida Plavec, Ales Berlec, Petr Maly, Milan Raska

**Affiliations:** a Department of Immunology, Faculty of Medicine and Dentistry, Palacky University Olomouc, Olomouc, Czech Republic; b Institute of Clinical and Molecular Pathology and Medical Genetics, University Hospital Ostrava and Faculty of Medicine, University of Ostrava, Ostrava, Czech Republic; c Institute of Clinical and Molecular Pathology, Faculty of Medicine and Dentistry, Palacky University Olomouc, Olomouc, Czech Republic; d Laboratory of Ligand Engineering, Institute of Biotechnology of the Czech Academy of Sciences, BIOCEV Research Center, Vestec, Czech Republic; e Department of Biotechnology, Jozef Stefan Institute, Ljubljana, Slovenia; f Faculty of Pharmacy, University of Ljubljana, Ljubljana, Slovenia

**Keywords:** IL-23, small binding protein, experimental colitis, recombinant *Lactococcus lactis*, oral therapy

## Abstract

Interleukin 23 (IL-23) is a key mediator of immune-mediated intestinal inflammation and plays an important role in the pathogenesis of inflammatory bowel disease. Inhibition of IL-23 signaling has therefore been validated as a therapeutic target in IBD. Here, we tested small binding proteins of non-immunoglobulin origin targeting either the IL-23 cytokine p19 subunit or the IL-23 receptor subunit IL-23R, designated ILP and REX binders, respectively. ILP317 and REX115 variants of formerly identified protein collections were tested in murine experimental colitis. *Lactococcus lactis* bacteria engineered for ILP317 or REX115 expression were administered to experimental mice via yogurt-coated food pellets before and during dextran sulfate sodium (DSS) and trinitrobenzenesulfonic acid (TNBS)-induced colitis. To compare both protein blockers, ILP317 and REX115 binders were administered intraperitoneally as soluble recombinant proteins. General health status, colon macroscopic and histological changes, mucosal tissue cell infiltration, local and systemic inflammatory cytokines were analyzed. In DSS- and TNBS-induced colitis, intraperitoneal administration of soluble ILP317 or REX115 reduced disease severity, including disease activity index, colon shortening, and mucosal inflammatory cell infiltration. Oral administration of recombinant *L. lactis* expressing ILP317 also showed consistent protective effects, whereas oral *L. lactis* expressing REX115 produced less pronounced effects in several experimental settings. Overall, ILP317 appeared more effective than REX115 when delivered orally by recombinant *L. lactis*. Oral administration of non-colonizing *L. lactis* expressing an IL-23p19 inhibitor may represent a promising preclinical strategy for the development of novel, non-invasive approaches to modulate intestinal inflammation.

## KEY FINDINGS

Interleukin-23 plays a major role in driving harmful gut inflammation, and blocking it can help ease inflammatory bowel disease. In this study, we tested two small proteins that interfere with IL-23 activity—either by blocking the IL-23 molecule itself or its receptor—and delivered them to mice either directly or using engineered *Lactococcus lactis* bacteria mixed into food. The treatments reduced intestinal inflammation and disease signs, suggesting that using *L. lactis* to deliver IL-23 blockers could offer a gentle, non-invasive future therapy for IBD.

## 1. Introduction

Inflammatory bowel disease (IBD) is a chronic immune-mediated inflammatory disorder of the gastrointestinal tract, characterised by persistent intestinal inflammation and tissue injury. Its pathogenesis involves the coordinated activity of innate immune cells, as well as T and B lymphocytes (Xavier and Podolsky 2007). IBD includes Crohn’s disease (CD), ulcerative colitis (UC), and indeterminate colitis (Geremia and Jewell 2012). Elevated levels of IL-23 are considered a key driver in IBD development (Friedrich et al. 2019). Increased IL-23 level has been associated with enhanced inflammatory responses in several murine colitis models, including dextran sulphate sodium (DSS)- and trinitrobenzenesulfonic acid (TNBS)-induced colitis (Cox et al. 2012; Wang et al. 2016). Under non-diseased conditions, IL-23 contributes to gut epithelial barrier integrity, limits epithelial permeability, and supports mucosal healing, partly by stimulating Th17 and innate lymphoid cells 3 (ILC3) to release cytokines such as IL-17 and IL-22 (Verstockt et al. 2023).

In murine models of colitis, IL-23 is produced by mucosal resident and inflammatory monocyte/macrophages, as well as by intestinal epithelial cells, in response to diverse triggers, including signals from commensal and pathogenic microbiota, activated dendritic cells, and damaged intestinal epithelial cells (Uhlig et al. 2006; Ivanov et al. 2009; Cox et al. 2012; Macho-Fernandez 2015; Fung et al. 2016). IL-23 is essential for the induction of murine intestinal inflammation, which is mediated by both innate and adaptive immune mechanisms. Global deletion of IL-23 or its receptor resulted in resistance to autoimmune and inflammatory disorders in many murine models (Cox et al. 2012). High susceptibility to murine TNBS-induced colitis was also associated with IL-23p19 deficiency. Thus, IL-23 exhibits not only pro-inflammatory, but also anti-inflammatory receptor-mediated effects in a context dependent manner (Oppmann et al. 2000; Becker et al. 2006). The IL-23 receptor is a complex of IL-12Rβ1 and IL-23R (IL-23Rα) subunits. The IL-23R subunit has been identified as an important susceptibility gene in Crohn’s disease by genome-wide association studies (Duerr et al. 2006; Van Limbergen et al. 2009; Sewell and Kaser 2022). IL-23R is expressed and signals in many cells including Th17 cells, CD8^+^ T cells, γδ T cells, macrophages, dendritic cells, intestinal epithelial cells, and ILC3.

In our study, we used two colitis models - DSS- and TNBS- induced colitis. DSS is a water-soluble, negatively charged sulfated polysaccharide that directly disrupts the epithelial integrity of the colon, leading to the development of severe colitis that most closely resembles human UC (Okayasu et al. 1990). TNBS is administered in an ethanol solution, where ethanol disrupts the intestinal barrier integrity and facilitates the interaction of haptenating TNBS with autologous colon tissue proteins or microbiota-derived proteins, resulting in significant activation of innate and T cells-mediated immune responses (Morris et al. 1989). In both DSS- and TNBS-induced colitis, disruption of epithelial barrier integrity promotes the release of damage-associated molecular patterns (DAMPs) and increases exposure of mucosal immune cells to microbial signals. DAMPs generated during epithelial injury activate intestinal epithelial cells, resident phagocytes, dendritic cells, and infiltrating myeloid cells, leading to enhanced production of pro-inflammatory cytokines such as IL-1β, IL-18, TNF-*α*, IL-6, IL-12, and IL-23 (Kontoyiannis et al. 1999; Siegmund et al. 2001; Sedhom et al. 2013; Hunter and Jones 2015). If this initial inflammatory response is not resolved, sustained production of these mediators supports recruitment and activation of innate immune cells and promotes pathogenic Th17-associated responses. In particular, prolonged IL-23 signalling contributes to the maintenance of pathogenic Th17 cells, epithelial damage, altered barrier integrity, and suppression of regulatory mechanisms, thereby amplifying intestinal inflammation. Thus, epithelial injury-driven cytokine induction in DSS- and TNBS-induced colitis provides a relevant experimental setting to evaluate therapeutic strategies targeting the IL-23/IL-23R axis. The importance of this pathway has been confirmed not only in these models, but also in other murine models of intestinal inflammation, where pharmacological inhibition of IL-23 or IL-23R reduced colitis activity (Kullberg et al. 2006; Elson et al. 2007; Cox et al. 2012; Guan et al. 2018).

Based on experimental colitis data, antibodies targeting IL-12p40 were tested and finally clinically approved for CD therapy (Feagan et al. 2017). In addition, monoclonal antibodies brazikumab, guselkumab, mirikizumab, and risankizumab targeting IL-23p19 were also approved for CD therapy (Feagan et al. 2017; Sands et al. 2017; McDonald et al. 2022).

Here, we used small binding proteins (binders) of non-immunoglobulin origin, targeting either the IL-23p19 subunit (binder ILP317) or IL-23R subunit (binder REX115) previously demonstrated as inhibitors of IL-23-mediated signalling using *in vitro* and *ex vivo* experiments (Kuchar et al. 2014; Krizova et al. 2017). Unlike conventional monoclonal antibodies, these small non-immunoglobulin binders represent a distinct class of targeting molecules with potential advantages, including reduced molecular size, engineering flexibility, and suitability for recombinant expression in microbial delivery systems. Their use therefore provides a novel strategy for local modulation of the IL-23 pathway directly within the gastrointestinal tract. To enable oral delivery, we generated recombinant *L. lactis* bacteria expressing either REX115 or ILP317 on their surface (Skrlec et al. 2018; Plavec et al. 2019) as a non-colonising, non-invasive system for delivery of active pharmaceutical ingredient to the gastrointestinal tract (Medici et al. 2025). Compared with systemic administration of purified proteins or monoclonal antibodies, this approach may allow transient local production and presentation of therapeutic binders in the intestinal lumen, thereby increasing mucosal exposure while potentially limiting systemic side effects. Moreover, recombinant *L. lactis* may help overcome major limitations associated with oral administration of protein-based therapeutics, particularly their reduced stability in the stomach and duodenum. Since the *L. lactis* prepared by our team can produce binders *in vivo* and the binders are stable, our blockers have a great potential for *per os* administration, as they can overcome the obstacles associated with the limited stability of proteins in the stomach and duodenum after oral administration. This strategy is particularly relevant in the context of the dual role of IL-23 in the intestine. Under homoeostatic conditions, IL-23 can contribute to epithelial barrier integrity, mucosal repair, and host defence. However, excessive or dysregulated IL-23 signalling promotes inflammatory responses and has been implicated in the pathogenesis of IBD and experimental colitis. Therefore, local and transient inhibition of the IL-23 pathway may provide a more targeted approach to reduce pathogenic inflammation while minimising broad systemic interference with IL-23-associated protective functions.

We compared the effectiveness of *per os* administration of a yogurt containing *L. lactis* bacteria expressing ILP317 or REX115 binders with intraperitoneal (*i.p.*) administration of respective ILP317 or REX115 proteins. We conclude that *i.p.* administered ILP317 or REX115 proteins are able to mitigate DSS- or TNBS-induced colitis by reducing colon tissue inflammation. Furthermore, oral administration of *L. lactis* expressing ILP317 demonstrated comparable efficacy, supporting recombinant *L. lactis*-mediated delivery of small IL-23-targeting binders as a promising preclinical strategy for the development of non-invasive approaches to modulate intestinal inflammation.

## 2. Methods

### 2.1. Bacterial cultivation and protein expression

*L. lactis* strains containing pSD-REX115-FLAG and pSD-ILP317-Myc (Zadravec et al. 2015; Skrlec et al. 2018; Plavec et al. 2019; Plavec et al. 2023) were grown in M17 medium (Merck, Darmstadt, Germany) supplemented with 0.5% glucose and 10 µg/ml chloramphenicol (GM17) without agitation at 30 °C. Protein expression was induced with 25 ng/ml of nisin (Merck) (Berlec and Strukelj 2009; Berlec et al., 2012Berlec et al. 2017) for 3 hours at an optical density A_600_ = 0.50. Bacterial cultures, both nisin-induced and non-induced, were stored at 4 °C for up to 5 days. Protein expression was monitored by western blot using a mouse monoclonal anti-FLAG tag antibody (1:1,000) (GenScript Biotech, NJ, U.S.A.) for REX115-FLAG and an anti-Myc tag mouse monoclonal antibody, clone 4A6 (1:1,000) (Merck), for ILP317-Myc. Proteins were visualised using a horseradish peroxidase-conjugated goat anti-mouse Ig antibody (1:1,000) (BD Pharmingen, Franklin Lakes, NJ, U.S.A.). Images were captured using a SynGene G:BOX Chemi XX9 device (Syngene, Cambridge, UK).

### 2.2. Flow cytometry analysis of *L. lactis* expressing ILP317 or REX115 isolated from pellets

One stool pellet was collected from each mouse directly into a tube, soaked in PBS for 20 min, resuspended, and centrifuged at 300 × g for 10 min. Subsequently, 100 μl of the supernatant was transferred to a new tube and centrifuged at 6,000 × g for 10 min. The resulting pellets were washed with PBS and stained with anti-Myc tag FITC antibody, clone 9E10 (Santa Cruz Biotechnology, TX, U.S.A.), for detection of *L. lactis* expressing ILP317 (*L. lactis* ILP317), or with anti-FLAG tag FITC antibody, clone OctA-Probe H-5 (Santa Cruz Biotechnology), for detection of *L. lactis* expressing REX115 (*L. lactis* REX115). Samples were analysed using a SONY SP6800 Spectral Analyser (Sony Biotechnology Inc., CA, U.S.A.).

### 2.3. Ethical approval statement and license numbers, experimental design

Animals were housed in the animal facility of the Faculty of Medicine and Dentistry, Palacký University Olomouc, which is approved by the Ministry of Agriculture of the Czech Republic for the housing and handling of laboratory animals (approval No. MZE-45559/2022-13143, valid until October 11, 2027). Animals were maintained in accordance with the ARRIVE guidelines and institutional animal care standards. All experimental protocols were approved by the Ethics Committee of the Faculty of Medicine and Dentistry, Palacký University Olomouc, and by the Ministry of Education, Youth and Sports of the Czech Republic under animal license number MSMT-10947/2021-3. All experiments were conducted in accordance with EU Directive 2010/63/EU on the protection of animals used for scientific purposes.

Eight- to nine-week-old female C57BL/6 or BALB/c mice (AnLab, Prague, Czech Republic), weighing 18–22 g before treatment, were maintained under standardised conditions at 21–22 °C, with a 12/12-h light/dark cycle and ad libitum access to food and water. Mice were allowed to acclimatise for 1 week before the start of the experiments (**Table S2 and S3).** Sample size justification and power analysis are provided in the Supplementary Materials. Animals were randomly assigned to experimental groups at the beginning of the study. Inclusion and exclusion criteria were established a priori. Animals were excluded from the colon analysis if they died during the study or required premature humane euthanasia due to welfare considerations or clinical necessity. No individual data points were excluded from the analysis unless valid measurement of the primary endpoint was not possible. To minimise bias during data acquisition and analysis, each mouse was identified only by a unique numerical code throughout the experiment. All downstream analyses, including clinical evaluation, histological assessment, cytokine measurements, and flow cytometry analyses, were performed using these coded identifiers without knowledge of the corresponding treatment groups. Group allocation was revealed only after completion of data analysis.

### 2.4. Induction, termination, and sample processing in mouse models of acute colitis

**DSS-induced colitis.** Acute DSS-induced colitis was induced in C57BL/6 mice by oral administration of 2.5% (w/v) DSS (MW approximately 40 kDa; TdB Labs, Uppsala, Sweden) in drinking water for 4 days.

**TNBS-induced colitis.** BALB/c mice were sensitised 7 days before TNBS colitis induction by epicutaneous application of 100 µl of 2.5% TNBS (Merck) in 50% EtOH onto shaved abdominal skin. Colitis was induced under anaesthesia with ketamine/xylazine (100 mg/kg ketamine and 5 mg/kg xylazine, i.p.; Bioveta, Ivanovice na Hané, Czech Republic) by rectal administration of 100 µl of 2.5% TNBS in 50% EtOH. Two days later, the experiment was terminated.

**Sample collection.** Mice were euthanized by cervical dislocation under ketamine/xylazine anaesthesia. Blood was collected, and the colon was removed, photographed, and its length from the caecum to the proximal rectum was measured. The terminal colon was dissected for histochemistry, quantitative real-time RT-PCR (qRT-PCR), and immune cell quantification by flow cytometry (**Figure S1**).

### 2.5. Oral administration of *L. lactis*

Preparation of food for one mouse: 2 × 10^9^ CFU of recombinant or wild type *L. lactis* were mixed with 3 g of white yogurt (2.7% fat; OLMA, Olomouc, Czech Republic) supplemented with 100 ng of nisin. 5 g of regular chow were coated with prepared yogurt mixture. Mice were housed in groups of three or four animals per cage (for a total of 10 mice per experimental group). In combination with ad libitum access to a sufficient amount of food supplemented with yogurt, this housing arrangement ensured comparable access to diet intake among animals and minimised variability in oral intake between mice. A freshly prepared mixture was provided to the mice every morning.

In two experimental groups, *L. lactis* expressing ILP317 or REX115 was administered by gavage at 2 × 10^9^ cells per mouse as a single daily dose.

### 2.6. Administration of ILP317, REX115 binders

Mice were injected intraperitoneally with 25 μg of binder (REX115 or ILP317) diluted in 100 μl of sterile PBS daily. The selected doses of ILP317 and REX115 were based on our previous studies describing the inhibitory effects of binders targeting IL-22 (Kuchař et al. 2024) and IL-6 (Groza et al. 2024). In these studies and the applied binder concentration (25 µg/mouse/dose) consistently demonstrated effective pathway inhibition and biological activity; therefore, the same concentration range was used in the present experiment.

### 2.7. Disease activity index calculation

Disease Activity Index calculation (DAI) describing body weight decrease, stool consistency, and rectal bleeding was calculated as reported by Morris et al. (1989) Table 1. DAI scores were used to evaluate the severity of DSS- and TNBS-induced colitis. The DAI index was calculated as a sum of scores for body weight decrease, stool consistency, and rectal bleeding.

**Table 1.** Calculation of DAI index using sum of scores for body weight decrease, stool consistency, and rectal bleeding (Morris et al. 1989).

Score	Body weight decrease	Stool consistency	Rectal bleeding
0	<1%	Normal	Normal
1	1–5%	Light loose	Light surface positive
2	6–10%	Loose stools	–
3	11–15%	Light diarrhoea	Bleeding
4	>15%	Diarrhoea	Gross bleeding

### 2.8. Measurement of serum cytokine concentration

The concentration of cytokines IL-2, IL-10, IL-12p40, IL-13, IL-17A, IL-18, IL-22, IL-23 and IFN-*α* in serum was measured using fluorescent microbeads (LEGENDplex™ Custom Mouse Assay, Biolegend, CA, U.S.A.) conjugated with the particular antibody according to the manufacturer ´ s instructions. The beads were analysed using a spectral cell analyser SONY SP6800 (Sony Biotechnology Inc, CA, U.S.A.).

### 2.9. RNA isolation and reverse transcription quantitative PCR (RT-qPCR)

Colon tissues were stored in RNAlater (Invitrogen, ThermoFisher Scientific, MA, U.S.A.) until RNA extraction. Pre-weighed samples were homogenised using QIAshredder (Qiagen, Hilden, Germany), and total RNA was extracted using RNeasy Plus Mini Kit (Qiagen). Column DNase treatment (gDNA Eliminator spin column, Qiagen) was used to eliminate potential DNA contamination. RNA purification was performed using a lithium chloride method (Juritsch and Moreau 2019) to avoid inhibitory activity of traces of DSS. qRT-PCR (Gb SG PCR Master Mix, Generi Biotech) was performed in triplicates according to the manufacturer's protocol on LightCycler® 480 System (Roche, Basel, Switzerland). All primers used in this study are listed in **Table S1**. Ct values were normalised to the reference gene, *GAPDH*, and the relative RNA transcription was calculated by the 2^−ΔΔCt^ method (Livak and Schmittgen 2001).

### 2.10. Histology

Colon tissues were fixed in 10% neutral-buffered formalin (Merck), stained with hematoxylin and eosin (H&E; Merck), and evaluated by a pathologist blinded to the clinical parameters. Tissue histology was examined using a BX43 microscope equipped with a CCD camera (Olympus, Tokyo, Japan). Tissue samples were scored for inflammatory cell infiltration, mucosal architecture, and epithelial changes according to Erben et al. (2014), as summarised in Table 2 and 3. Necrosis was classified as focal when involving ≤15% of the affected tissue (score 10) and diffuse when involving >15% of the affected tissue (score 20).

**Table 2.** Scoring scheme for inflammatory cell infiltrate in chemically induced colonic inflammation.

Inflammatory cell infiltrate:		
Severity	Extent	Score
Minimal	Mucosa	1
Mild	Submucosa	2
Moderate	Transmural < 50%	3
Marked	Transmural > 50%	4

**Table 3.** Scoring scheme for intestinal architecture in chemically induced colonic inflammation.

Intestinal architecture			
Epithelial changes	Score	Mucosal architecture	Score
Hyperplasia	1–5	Ulceration	4–5
Goblet cell loss	1–5	Granulation	4–5
Cryptitis	2–3	Irregular crypts	4–5
Crypt abscesses	3–5	Crypt loss	4–5
Erosion	1–4	Villous blunting	1–5
Necrosis	10,20		

### 2.11. Flow cytometry analysis of intestinal cells

Intestinal tissues were pre-incubated in a pre-digestion solution consisting of Hank’s Balanced Salt Solution supplemented with 4% foetal bovine serum (FBS) and 5 mM EDTA, followed by incubation in a digestion solution consisting of D-PBS with Ca^2+^ and Mg^2+^, 0.1% collagenase D, 50 U/ml DNase, and 4% FBS. Surface Fc receptors were blocked with TruStain FcX antibody (BioLegend) for 10 min, followed by surface staining with antibodies against CD3-BV785, CD4-BV650, CD90.2-Pacific Blue, CD19-BV570, CD11b-BV750, CD206-PerCP-Cy5.5, CD163-BV421, Ly-6C-BV711, Ly-6G-PE-Cy5, and CD170-PE-Dazzle594 (BioLegend), together with FVD-eFluor506 viability dye (eBioscience, Thermo Fisher Scientific). After permeabilization, intracellular staining was performed using GATA-3-PE-Cy7 and RORγT-PE antibodies (BD Biosciences). Samples were analysed using a SONY SP6800 Spectral Analyser (Sony Biotechnology), and the resulting FCS files were processed using FlowJo 10.10 software (BD Biosciences).

### 2.12. Statistical analysis

All statistical analyses were performed using GraphPad Prism 8 Software (GraphPad Software Inc., CA, U.S.A.). Statistical differences among multiple independent groups were analysed using the Kruskal–Wallis test followed by Dunn’s multiple comparisons test. The analyses were based on ranks, and adjusted *P* values were taken directly from the GraphPad Prism output. Prism reported approximate rather than exact *P* values for the Kruskal–Wallis analyses. Tied values and small group sizes were handled according to the standard rank-based procedures implemented in GraphPad Prism. A value of *p* < 0.05 was considered statistically significant. Assumptions for the Kruskal–Wallis test were considered met, as observations were independent, animals were randomly assigned to groups, and the distributions showed comparable spread across groups.

## 3. Results

### 3.1. Binding affinity of ILP317 and REX115 to murine IL-23 and IL-23R

Albumin-binding domain (ABD)-derived small binding proteins were originally selected to target either the human p19 subunit of IL-23 or the human IL-23 receptor as part of the IL-23 receptor signalling complex. The cDNA sequences encoding ILP317 and REX115 were subsequently used to generate genetically modified *L. lactis* strains secreting or surface-displaying the binders individually (Skrlec et al. 2018; Plavec et al. 2019) or concomitantly (Plavec et al. 2023). Since the present study evaluates these binders in murine models of experimentally induced colitis, we first assessed whether ILP317 and REX115 also interact with the corresponding murine targets.

The binding of ILP317 to murine IL-23 was evaluated using an ELISA-based binding assay. As shown in **Figure S2A**, ILP317 bound to immobilised murine recombinant IL-23-Fc-IgG in a concentration-dependent manner, whereas only minimal signal was detected in BSA-blocked control wells. The apparent Kd was estimated to be in the range of 30–40 nM, supporting specific binding of ILP317 to murine IL-23 and confirming its suitability for further evaluation in murine colitis models.

To validate REX115 binding to murine IL-23R, binding affinity and kinetics were measured using NIH-3T3 murine fibroblast cells and a LigandTracer Green Line instrument, which enables real-time monitoring of interactions between fluorescently labelled ligands and cell-surface targets. Based on kinetic analysis, the Kd of REX115 binding to murine IL-23R-expressing NIH-3T3 cells was calculated to be approximately 0.1 nM. For comparison, we also analysed binding to COS-7 cells, an African green monkey kidney fibroblast-like cell line, since monkey IL-23R shares high sequence identity with human IL-23R. REX115 binding to COS-7 cells was also detected, although the binding signal was lower than that observed with NIH-3T3 cells, with an estimated Kd of approximately 1 nM. Together, these results demonstrate high-affinity interaction of REX115 with murine IL-23R and support its use in murine in vivo models of colitis **(Figure S2B).**

### 3.2. Stability of ILP-, REX- expressing recombinant *L. lactis* bacteria *in vitro* and *in vivo*

IL-23 signalling inhibition, production of ILP and REX binders, and their modes of administration are schematically presented in Figure 1. Protein binders ILP317 and REX115 used for intraperitoneal administration were produced in *Escherichia coli* BL21 as 38 kDa fusion proteins and purified as previously reported (Kuchar et al. 2014; Krizova et al. 2017). *L. lactis* ILP317 and *L. lactis* REX115 used for oral administration were cultivated and prepared as described in the Methods section (Skrlec et al. 2018; Plavec et al. 2019). Yogurt served as a vehicle for oral administration of the recombinant *L. lactis* bacteria. The potential contribution of intrinsic yogurt bacteria on colitis course was considered by using an appropriate control (*L. lactis* producing no binders in yogurt designated as *L. lactis* ‘Wild’). During the experiment, the viability of the binder-producing bacteria *L. lactis* was not affected after 24 hours of storage at 4 °C or 22 °C (**Figure S3**). To confirm the intra-intestinal viability of *L. lactis* and the delivery of binders, we isolated recombinant *L. lactis* from faecal pellets and verified its viability by *in vitro* cultivation for at least 11 h after chow administration containing *L. lactis* (**Figure S4**). We further assessed the presence of recombinant ILP317 and REX115 proteins on the surface of *L. lactis* isolated from faecal samples by flow cytometry (Figure 2). These analyses confirmed both the viability of *L. lactis* and the stability of ILP317 and REX115 surface expression within the intestinal tract under both DSS- and TNBS-treatment conditions.

**Figure 1.**
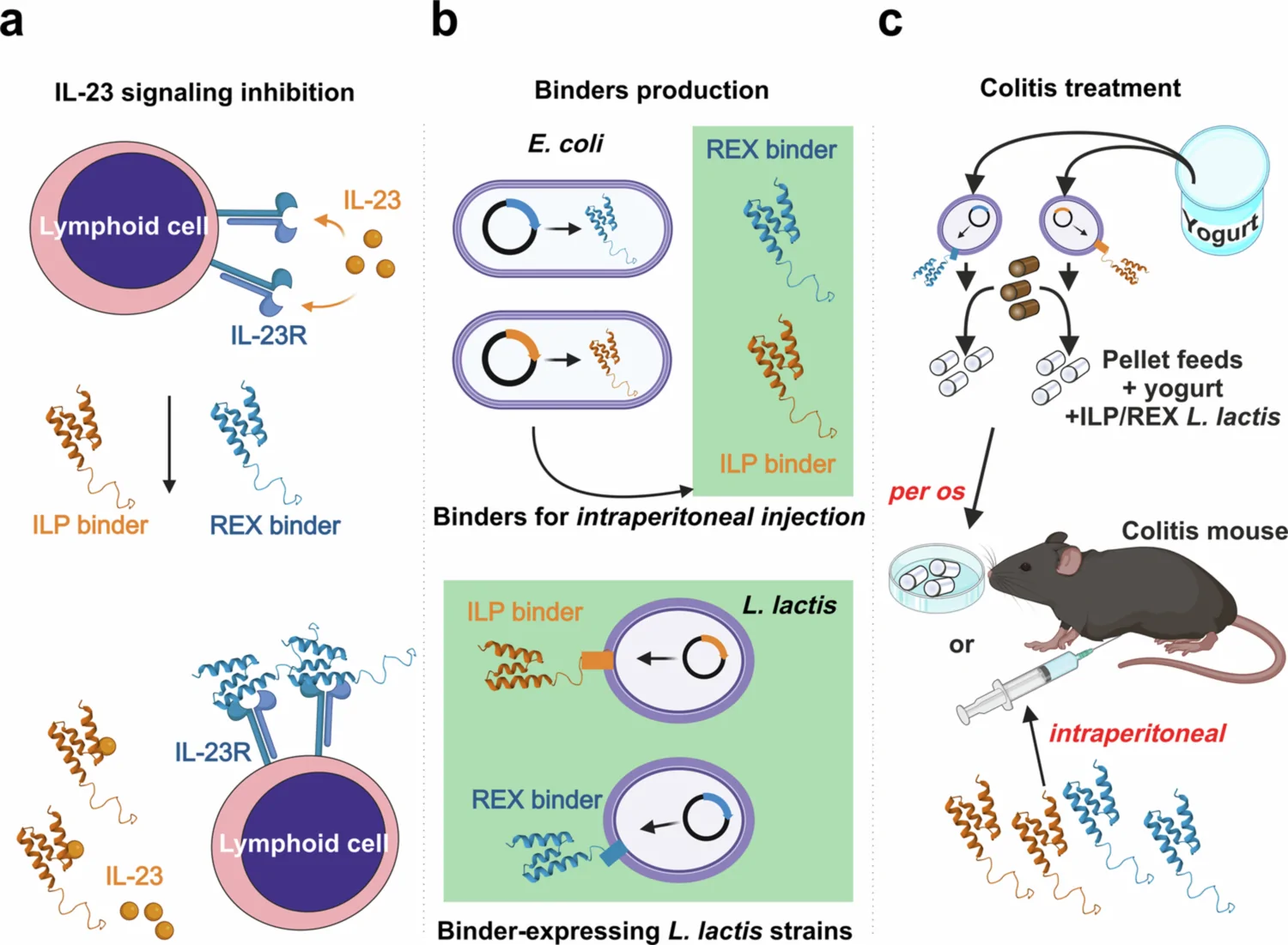
Characterization, production, and administration routes of ILP and REX binders in experimental colitis. **a)** Schematic representation of IL-23 pathway targeting by the non-immunoglobulin binders used in this study. The ILP binder targets the IL-23 cytokine, whereas the REX binder targets the IL-23 receptor. **b)** Binder production was performed using two recombinant expression systems. *Escherichia coli* was used to produce purified ILP and REX binders for intraperitoneal administration, whereas *Lactococcus lactis* was engineered to produce binder-expressing strains displaying ILP or REX binders on the bacterial surface for oral delivery. **c)** Experimental administration strategy in the DSS-induced mouse model of colitis. Purified ILP and REX binders were administered intraperitoneally, whereas recombinant *L. lactis* expressing the corresponding binders was administered orally in yogurt applied to food pellets. The figure was created with BioRender.com.

**Figure 2.**
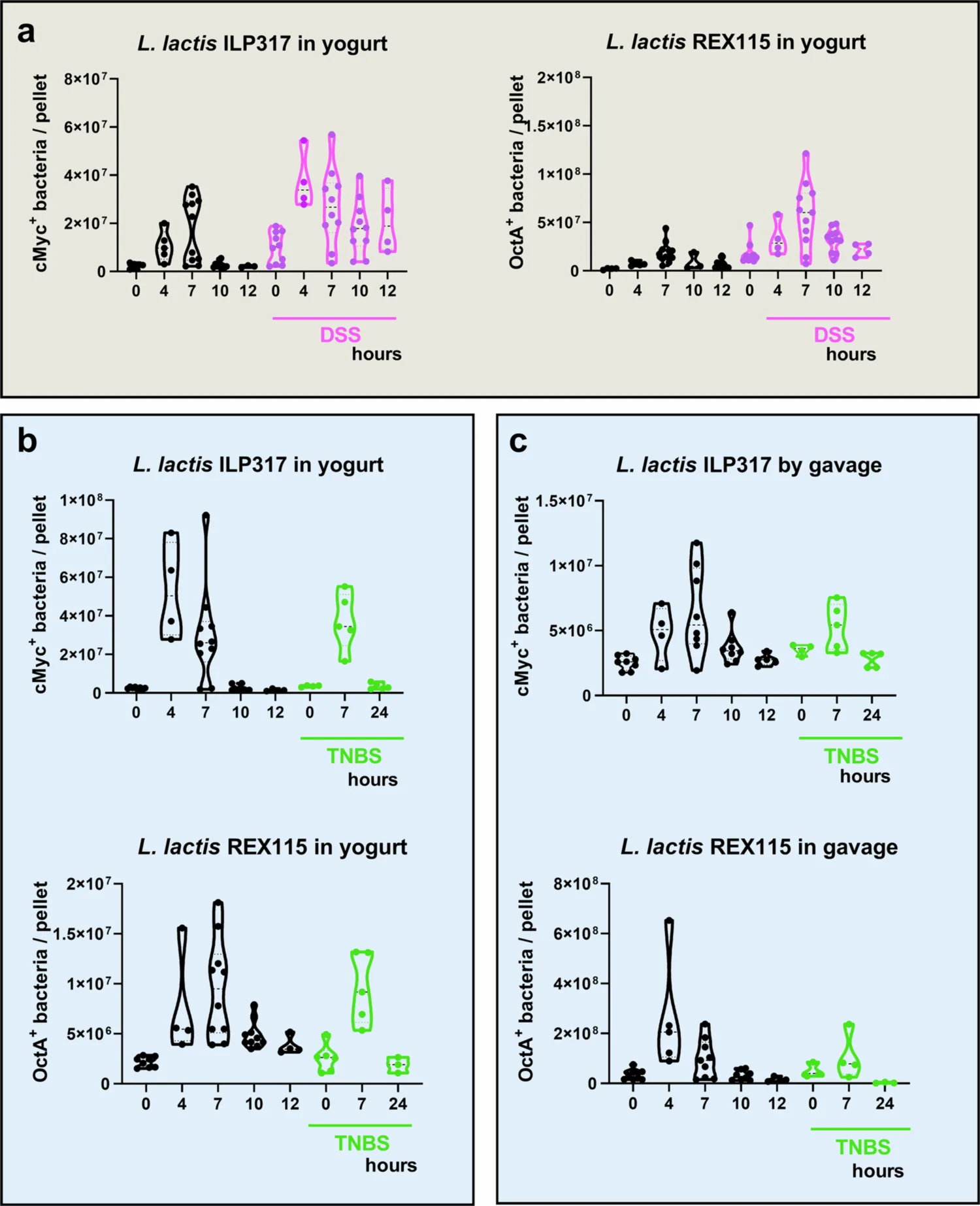
Time course of the presence of *L. lactis* expressing ILP317 or REX115 in the stool of mice in experimental DSS or TNBS-induced colitis. Detection of recombinant *L. lactis* bacteria expressing ILP317 or REX115 in stool samples from mice receiving yogurt containing the corresponding recombinant *L. lactis* strain during **a)** DSS-induced colitis and **b)** TNBS-induced colitis. ILP317 and REX115 surface expression was detected using anti-Myc and anti-OctA antibodies, respectively, and analysed by flow cytometry. Data are expressed as the number of positive bacteria per stool pellet. **c)** Detection of ILP317- or REX115-expressing *L. lactis* in stool samples from mice receiving the corresponding recombinant *L. lactis* strain by gavage during TNBS-induced colitis. The difference in timepoints reflects experimental constraints and sampling feasibility inherent to each model. In the DSS model (oral administration), mice maintain sufficient stool production, enabling sampling across extended timepoints (0, 4, 7, 10, 12 h) to monitor the persistence of *L. lactis* in the gut. In contrast, the TNBS model, which involves epicutaneous sensitisation followed by intrarectal TNBS administration under anaesthesia, induces a more acute and severe form of colitis. As a result, mice show reduced food intake and limited stool production shortly after induction. Stool collection was therefore technically more limited than in the DSS model, allowing reliable assessment of *L. lactis* in stool only at early timepoints (0, 7, and 24 h). For the DSS experiment, group sizes were as follows: *L. lactis* ILP317, *n* = 10; *L. lactis* REX115, *n* = 10. For the TNBS experiment, group sizes were as follows: *L. lactis* ILP317, *n* = 10; *L. lactis* REX115, *n* = 10; gavage *L. lactis* ILP317, *n* = 9; and gavage *L. lactis* REX115, *n* = 9. Data are shown as box-and-whisker plots with whiskers indicating minimum and maximum values. No statistical analysis was performed for this figure.

### 3.3. ILP317 and REX115 protein binders significantly alleviate clinical and histomorphological manifestations of DSS- and TNBS-induced colitis

In the DSS-induced colitis model, binders were administered for 7 consecutive days, starting three days prior to colitis induction (Figure 3a). Female C57BL/6 mice received binders either intraperitoneally as a recombinant protein solution (25 μg in sterile PBS, once daily) or orally as *L. lactis* ILP317 or *L. lactis* REX115 cells (2 × 10^9^ CFU daily per mouse) delivered in yogurt coated onto standardised mouse chow.

**Figure 3.**
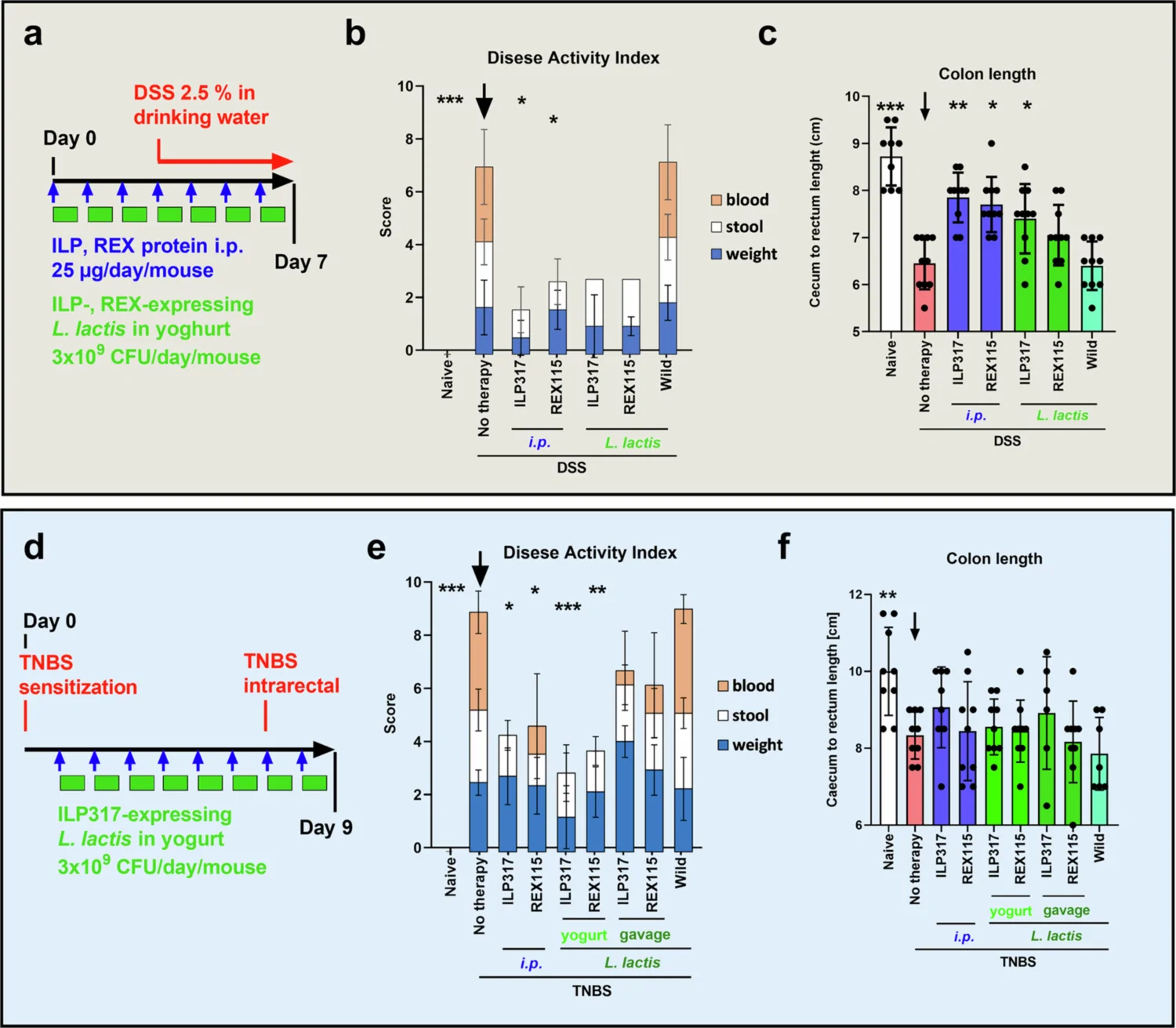
Clinical effects of preventive-therapeutic ILP and REX treatment in DSS- and TNBS-induced colitis models **a)** Experimental scheme of DSS-induced colitis. Preventive-therapeutic administration of ILP317 or REX115 started on day 0, either intraperitoneally as purified recombinant proteins or orally as recombinant *L. lactis* expressing the corresponding binder in yogurt-coated food pellets, while colitis was induced from day 3 by administration of 2.5% DSS in drinking water. **b)** Disease activity index (DAI) in DSS-induced colitis, calculated as the cumulative score of body weight loss, stool consistency, and rectal bleeding. The cumulative DAI score was used for statistical evaluation. **c)** Colon length measured after euthanasia in DSS-induced colitis groups as a macroscopic indicator of disease severity. **d)** Experimental scheme of TNBS-induced colitis and treatment administration relative to TNBS induction. **e)** DAI score in TNBS-induced colitis, calculated as the cumulative score of body weight loss, stool consistency, and rectal bleeding. The cumulative DAI score was used for statistical evaluation. **f)** Colon length measured after euthanasia in TNBS-induced colitis groups. For the DSS experiment, group sizes were as follows: Naïve, *n* = 9; No therapy, *n* = 10; *i.p.* ILP317, *n* = 10; *i.p.* REX115, *n* = 10; *L. lactis* ILP317, *n* = 10; *L. lactis* REX115, *n* = 10; and *L. lactis* Wild, *n* = 10. For the TNBS experiment, group sizes were as follows: Naïve, *n* = 10; No therapy, *n* = 10; *i.p.* ILP317, *n* = 9; *i.p.* REX115, *n* = 9; *L. lactis* ILP317, *n* = 10; *L. lactis* REX115, *n* = 10; *L. lactis* Wild, *n* = 10; gavage *L. lactis* ILP317, *n* = 9; and gavage *L. lactis* REX115, *n* = 9. Statistical differences were analysed by Kruskal-Wallis one-way ANOVA model followed by Dunn ´ s multiple comparisons test. Statistical significance is shown relative to the No therapy group. Means + /- SD are shown. *P < 0.05, **P < 0.01, ***P <0.001.

Before termination of the murine experiment, the DAI score was assessed (Figure 3b). Following euthanasia, the colon was dissected, photographed (**Figure S5**), and its length measured (Figure 3c).

C57BL/6 mice exposed to DSS (No therapy group) or treated with yogurt containing *L. lactis* wild (*L. lactis* Wild group) exhibited significantly increased DAI scores compared to Naïve group of animals (from 0 ± 0 in Naïve to 6.5 ± 0.5 in DSS No therapy or 6.4 ± 0.5 in DSS *L. lactis* Wild group) (Figure 3b). Intraperitoneal treatment with either ILP317 or REX115, as well as oral administration of *L. lactis* ILP317 or *L. lactis* REX115, reduced DAI scores from 2.3 ± 1.5 to 3.0 ± 1.3, depending on treatment, corresponding to an approximately 54–65% reduction compared with the DSS No therapy group. This reduction reached statistical significance in the intraperitoneally treated groups. Rectal bleeding, a typical feature of DSS-induced colitis observed in the No therapy and *L. lactis* Wild groups, was absent in all ILP317- and REX115-treated mice.

DSS-induced intestinal alterations were initially characterised by a reduction in colon length, from 9.1 ± 0.4 cm in Naïve mice to 6.5 ± 0.5 cm in both DSS No therapy and DSS *L. lactis* Wild groups. Consistent with the DAI scores, colon shortening was markedly attenuated in mice treated *i.p.* with REX115 or *i.p.* with ILP317, or orally with *L. lactis* ILP317. These differences in colon length were statistically significant compared to the DSS No therapy group, except for mice treated with *L. lactis* REX115 (Figure 3c).

For TNBS-induced colitis, binders were administered to BALB/c mice for 7 consecutive days, with the first dose given five days prior to colitis induction (Figure 3d). Binders were delivered using the same methods as in DSS-induced colitis—*i.p.* or orally as *L. lactis* recombinant ILP317 or REX115 cells in yogurt. As a control for the oral route in yogurt, *L. lactis* expressing ILP317 or REX115 were administered by gavage (2 × 10^9^ cells in a single daily dose).

The No therapy group and the *L. lactis* Wild group exhibited significantly increased DAI scores compared with Naïve mice, increasing from 0.0 ± 0 in the Naïve group to 7.6 ± 1.6 in the No therapy group and 7.7 ± 1.5 in the *L. lactis* Wild group. Both intraperitoneal administration of ILP317 or REX115 and oral administration of *L. lactis* ILP317 or *L. lactis* REX115 in yogurt significantly reduced DAI scores. After intraperitoneal delivery, DAI scores decreased to 48% and 52% of the No therapy group for ILP317 and REX115, respectively, whereas oral delivery via *L. lactis* in yogurt reduced DAI scores to 32% and 42% of the No therapy group for *L. lactis* ILP317 and *L. lactis* REX115, respectively (Figure 3e). Administration of *L. lactis* ILP317 or *L. lactis* REX115 by gavage resulted in a more moderate decrease in DAI scores, reaching 75% and 69% of the No therapy group, respectively. Anal bleeding, observed in the No therapy and *L. lactis* Wild groups, was absent in the i.p. ILP317 group and in the groups receiving oral *L. lactis* expressing ILP317 or REX115.

BALB/c mice exposed to TNBS exhibited significant colon shortening (10.0 ± 1.5 cm in the Naïve group vs. 8.3 ± 0.6 cm in the No therapy group and 7.9 ± 1.0 cm in the *L. lactis* Wild group). However, colon length did not differ significantly between any of the treatment groups and the No therapy group (Figure 3f).

Next, we evaluated the histomorphological changes in the large intestine mucosa in DSS-induced colitis (Tables 2 and 3) (Figure 4a, b, c). A significant reduction in inflammatory cells infiltration was observed following *i.p.* ILP317, *i.p.* REX115, and oral *L. lactis* ILP317 treatment. Intraperitoneal administration of ILP317 also significantly prevented deterioration of mucosal architecture (including ulcerations, granulation tissue, irregular crypts, or crypt loss), with severity scores comparable to those of Naïve mice (Figure 4c). Regarding epithelial changes—such as goblet cells loss, epithelial hyperplasia, cryptitis, and crypt abscesses—only modest alterations were induced by DSS in the No therapy group (Figure 4b). Representative pictures of H&E-stained colon sections are shown in Figure 4g.

**Figure 4.**
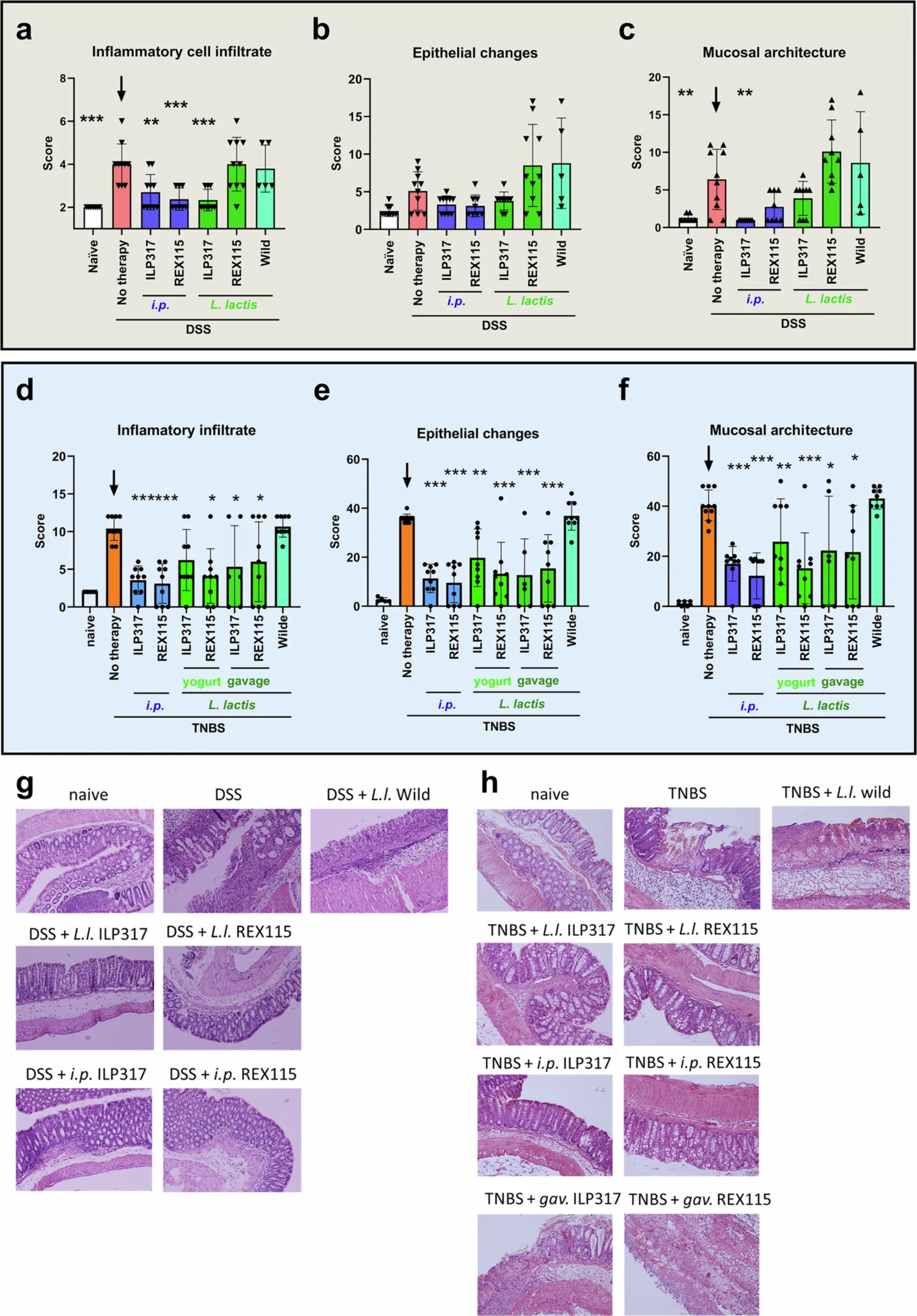
Histological evaluation of colon tissue in DSS- and TNBS-induced colitis. Histological changes in the distal third of the colon were assessed in H&E-stained sections according to the scoring system described by Erben et al. (43). In DSS-induced colitis, histological scoring included **a)** inflammatory cell infiltration, **b)** mucosal architecture, and **c)** epithelial changes. In TNBS-induced colitis, the same histological parameters were evaluated: **d)** inflammatory cell infiltration, **e)** mucosal architecture, and **f)** epithelial changes. These parameters reflect the extent of mucosal inflammation, disruption of tissue architecture, and epithelial injury induced by experimental colitis and modified by ILP317 or REX115 treatment. **g)** Representative H&E-stained colon sections from DSS-induced colitis showing characteristic histological alterations compared with normal colon tissue. Naïve—normal colon; No therapy—moderate inflammatory cell infiltrate, severe goblet cell loss, severe epithelial changes, distortion of mucosal architecture, hyperregenerative atypia; Wild *L. lactis* - moderate inflammatory cell infiltrate, severe goblet cell loss, severe epithelial changes, distortion of mucosal architecture, presence of ulceration, hyperregenerative atypia *i.p.* ILP317—normal colon; *i.p.* REX115 - minimal inflammatory cell infiltrate, minimal epithelial changes with well-preserved mucosal architecture; *L. lactis* ILP317 - minimal inflammatory cell infiltrate, minimal epithelial changes with well-preserved mucosal architecture; *L. lactis* REX115 - minimal inflammatory cell infiltrate, minimal epithelial changes with well-preserved mucosal architecture. **h)** Representative H&E-stained colon sections from TNBS-induced colitis: Characteristic alterations compared to normal histology are specified. Naïve—normal colon; No therapy—focal necrosis of mucosa, marked transmural inflammation, goblet cell loss, severe crypt loss, severe villous blunting, erosion, hyperplasia, crypt abscesses, severe ulceration; Wild *L. lactis*—focal necrosis, moderate transmural inflammatory cell infiltrate, severe goblet cell loss, severe epithelial changes, distortion of mucosal architecture, presence of ulceration, *i.p.* ILP317—mild submucosal inflammation, goblet cell loss, severe crypt loss, severe villous blunting, minor erosion, mild hyperplasia, crypt abscesses; *i.p.* REX115—normal colon; *L. lactis* ILP317—normal colon; *L. lactis* REX115—normal colon; gavage *L. lactis* ILP317 - diffuse necrosis of mucosa, goblet cell loss, marked transmural inflammation, several crypt loss, several villous blunting, mild hyperplasia, ulceration; gavage *L. lactis* REX115 - focal necrosis of mucosa, goblet cell loss, marked transmural inflammation, several crypt loss, several villous blunting, minimal hyperplasia, crypt abscesses, ulceration. For the DSS experiment, group sizes were as follows: Naïve, *n* = 9; No therapy, *n* = 10; *i.p.* ILP317, *n* = 10; *i.p.* REX115, *n* = 10; *L. lactis* ILP317, *n* = 10; *L. lactis* REX115, *n* = 10; and *L. lactis* Wild, *n* = 10. For the TNBS experiment, group sizes were as follows: Naïve, *n* = 10; No therapy, *n* = 10; *i.p.* ILP317, *n* = 9; *i.p.* REX115, *n* = 9; *L. lactis* ILP317, *n* = 10; *L. lactis* REX115, *n* = 10; *L. lactis* Wild, *n* = 10; gavage *L. lactis* ILP317, *n* = 9; and gavage *L. lactis* REX115, *n* = 9. Statistical differences were analysed by Kruskal-Wallis one-way ANOVA model followed by Dunn ´ s multiple comparisons test. Statistical significance is shown relative to the No therapy group. Means + /- SD are shown. *P < 0.05, **P < 0.01, ***P < 0.001.

TNBS-induced colitis was characterised by severe infiltration of inflammatory cells, predominantly in the submucosa and extending transmurally, accompanied by marked alterations in mucosal architecture (most pronounced in the scores for irregular crypts, crypt loss, and villous blunting) as well as significant epithelial changes (most severe in the scores for goblet cells loss, cryptitis, and crypt abscesses) (Figure 4d,e,f). The *i.p.* administration of ILP317 and REX115, as well as oral administration of yogurt containing *L. lactis* ILP317 or *L. lactis* REX115, led to significant suppression of all described histomorphological changes typical for TNBS- induced colitis, such as inflammatory cell infiltration, epithelial alterations, and mucosal architecture (Figure 4d,e,f). Administration of *L. lactis* ILP317 and *L. lactis* REX115 by gavage provided protection in some mice but not in others, and thus gavage of *L. lactis* expressing ILP317 or REX115 did not exhibit a significant therapeutic effect. In contrast, we observed a marked protective effect of *i.p.* administration of ILP317 and REX115, as well as oral application of *L. lactis* ILP317 or *L. lactis* REX115 in yogurt, against the development of necrosis and ulceration in response to TNBS. The extent of necrosis and the presence of ulceration are summarised in Figure 5. The No therapy group showed focal and marked focal necrosis. In contrast, both *i.p.* and oral administration of ILP317 and REX115 in yogurt suppressed the development of colonic necrosis. Consistently, the No therapy group exhibited ulceration, whereas *i.p.* administration of ILP317 and REX115 binders, as well as oral administration of ILP317- or REX115-expressing *L. lactis* in yogurt, protected the colon against ulceration. Administration of ILP317 and REX115 by gavage demonstrated only partial protective effects in some mice.

**Figure 5.**
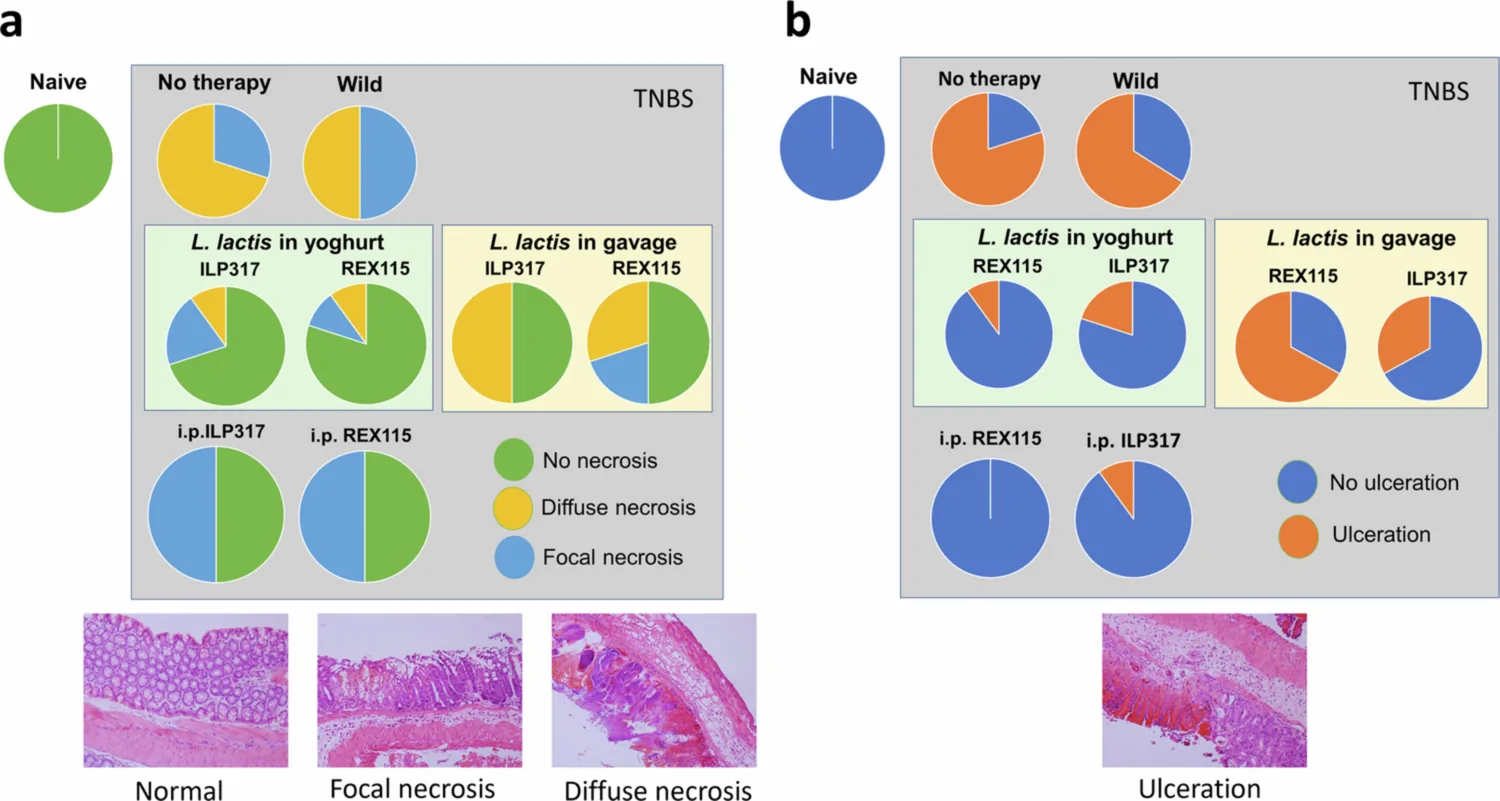
ILP317 and REX115 reduce necrosis and ulceration in TNBS-induced colitis. The two-panel figure summarizes the severity and distribution of destructive histopathological changes in TNBS-induced colitis using pie charts and representative H&E-stained colon sections. **a)** Evaluation of colonic necrosis across the TNBS-induced colitis treatment groups. TNBS treatment induced focal to diffuse necrotic changes in untreated mice, whereas ILP317 and REX115 treatment reduced the extent of necrosis. The protective effect was most pronounced after oral administration of *L. lactis* ILP317 and *L. lactis* REX115 in yogurt, while intraperitoneal treatment prevented the development of diffuse necrosis. **b)** Evaluation of ulceration across the TNBS-induced colitis treatment groups. Ulceration was observed in untreated TNBS colitis, whereas all therapeutic interventions reduced ulceration development. Intraperitoneal REX115 treatment completely abolished ulceration in this model. Representative histological images illustrate the corresponding tissue alterations and treatment-associated protection against necrosis and ulceration. Data are presented as histological scores/distribution categories. Group sizes were as follows: Naïve, *n* = 10; No therapy, *n* = 10; *i.p.* ILP317, *n* = 9; *i.p.* REX115, *n* = 9; *L. lactis* ILP317, *n* = 10; *L. lactis* REX115, *n* = 10; *L. lactis* Wild, *n* = 10; gavage *L. lactis* ILP317, *n* = 9; and gavage *L. lactis* REX115, *n* = 9.

In conclusion, we demonstrated that *i.p.* administration of ILP317 or REX115, as well as oral delivery of recombinant *L. lactis* expressing ILP317 or REX115 in yogurt, represent effective therapeutic approaches in both DSS- and TNBS-induced colitis.

### 3.4. ILP317 and REX115 reduce the gene expression of colitis-associated cytokines in colon tissue

DSS-induced colitis is associated with increased gene expression of cytokines in large intestinal segments. In the No therapy group, we confirmed significantly elevated gene expression of IL-6, IL-10, IL-22, IL-17A, IL-1β, IL-22BP, and TNF-*α* in colonic tissue, as determined by qRT-PCR (Figure 6, S6). By contrast, in the DSS *L. lactis* Wild group, transcript levels of IL-10, IL-17A, IL-1β, and TNF-*α* did not reach statistical significance compared with Naïve mice. These results indicate that oral administration of wild type *L. lactis* may partially attenuate DSS-induced immune activation in the colon (IL-10, IL-17A, IL-1β, and TNF-*α*), although the effect was not statistically significant relative to the DSS No therapy group.

The efficacy of REX115 and ILP317 treatments was further supported by marked suppression of IL-10 gene expression in DSS-induced colitis across all treatment groups (Figure 6a). Compared with the DSS No therapy group, IL-10 expression was reduced by approximately 95.4–98.5% following intraperitoneal administration of ILP317 or REX115 and oral administration of *L. lactis* ILP317 or *L. lactis* REX115. For IL-6, a statistically significant decrease compared with the DSS No therapy group was observed only following treatment with *L. lactis* ILP317, corresponding to an approximately 91% reduction, although a decreasing trend was also observed in the other treatment groups, including *L. lactis* REX115, *i.p.* REX115, and *i.p.* ILP317, with reductions of approximately 73.4%, 67%, and 59%, respectively (Figure 6b). IL-22 transcription was significantly reduced in the *i.p.* REX115 binder group and in mice treated orally with *L. lactis* expressing ILP317, corresponding to an approximately 89% reduction compared with the DSS No therapy group (Figure 6c). Gene expression of IL-22BP receptor was significantly reduced only in the *i.p.* REX115 group, corresponding to an 89% reduction compared with the DSS No therapy group. Expression levels of TNF-*α*, IL-17A, and IL-1β were not significantly affected by any of the treatments tested (**Figure S6**).

**Figure 6.**
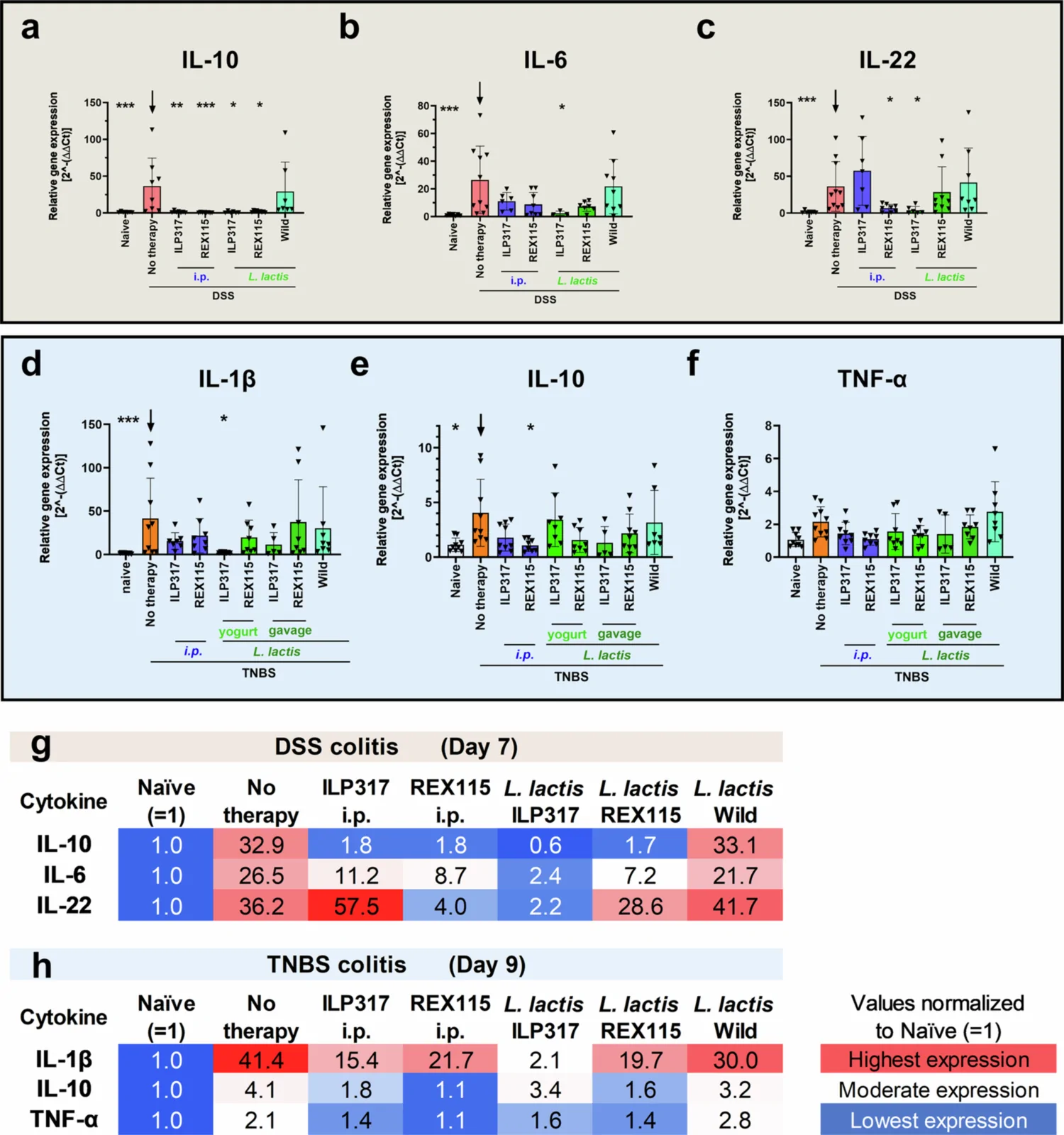
ILP317 and REX115 reduce colitis-associated cytokine gene expression in colon tissue. qRT-PCR analysis was performed to determine whether ILP317 and REX115 treatment modulated local cytokine responses in DSS- and TNBS-induced colitis. In DSS-induced colitis, colonic expression of **a)** IL-10, **b)** IL-6, and **c)** IL-22 was assessed as representative cytokines associated with mucosal inflammation and IL-23-related immune activation. In TNBS-induced colitis, expression of **d)** IL-1β, **e)** IL-10 and **f**) TNF*α* was analyzed to evaluate treatment-associated modulation of acute inflammatory responses. Expression of particular cytokine is expressed relative to the Naïve group in all graphs. **In DSS-induced colitis**, IL-10, IL-22, and IL-6 were significantly increased in the No therapy group. All treatments significantly reduced IL-10 gene expression compared to the No therapy group. *L. lactis* ILP317 significantly reduced IL-10, IL-6, and IL-22 expression relative to the No therapy group. **In TNBS-induced colitis**, IL-1β and IL-10 were significantly upregulated in the No therapy group compared to the naïve group. *L. lactis* ILP317 significantly decreased IL-1β expression, while *i.p.* REX115 significantly reduced IL-10 expression. All treatments, except *L. lactis* REX115 gavage, showed a clear but non-significant effect on IL-1β expression, whereas all treatments, except *L. lactis* ILP317 and *L. lactis* REX115 gavage, exhibited a clear but non-significant effect on IL-10. There was a mild impact of TNBS and used treatments on TNFα gene expression. **g,h)** Heatmaps summarising cytokine expression data presented in panels **(a–f)**. Values represent mean cytokine expression levels normalised to the naïve control group. Dark blue indicates the lowest relative cytokine expression, white indicates intermediate expression, and red indicates the highest relative cytokine expression. For the DSS experiment, group sizes were as follows: Naïve, *n* = 9; No therapy, *n* = 10; *i.p.* ILP317, *n* = 10; *i.p.* REX115, *n* = 10; *L. lactis* ILP317, *n* = 10; *L. lactis* REX115, *n* = 10; and *L. lactis* Wild, *n* = 10. For the TNBS experiment, group sizes were as follows: Naïve, *n* = 10; No therapy, *n* = 10; *i.p.* ILP317, *n* = 9; *i.p.* REX115, *n* = 9; *L. lactis* ILP317, *n* = 10; *L. lactis* REX115, *n* = 10; *L. lactis* Wild, *n* = 10; gavage *L. lactis* ILP317, *n* = 9; and gavage *L. lactis* REX115, *n* = 9. Statistical differences were analysed by Kruskal-Wallis one-way ANOVA model followed by Dunn ´ s multiple comparisons test. Statistical significance is shown relative to the No therapy group. Means + /- SD are shown. *P < 0.05, **P < 0.01, ***P < 0.001.

In TNBS-induced colitis, significantly elevated expression of IL-1β and IL-10 was observed in the colonic tissue of the No therapy group (Figure 6d, e). Oral administration of *L. lactis* ILP317 in yogurt significantly reduced IL-1β expression by up to 95% compared with the TNBS No therapy group, whereas all other treatments, except REX115 gavage, showed a clear but non-significant reduction. For IL-10, expression was significantly decreased following *i.p.* administration of REX115 by up to 73.5% compared with the TNBS No therapy group, whereas all other treatment groups, except *L. lactis* ILP317 in yogurt and *L. lactis* REX115 by gavage, exhibited a clear but non-significant trend toward reduced expression. However, IL-10 expression changes occurred within a relatively narrow range of relative gene expression values [2^−(ΔΔCt): 0–4], substantially lower than the dynamic range observed for IL-1β [2^−(ΔΔCt): 0–41]. Therefore, although IL-10 modulation was detectable, the magnitude of this change was relatively limited compared with the more pronounced alterations observed for IL-1β. No significant changes in TNF-*α* expression were observed in any of the tested groups.

Heatmaps summarising all mean cytokine expression values normalised to the Naïve control group are shown in Figure 6g,h.

Since ILP317 and REX115 are inhibitors of IL-23 signalling, we investigated whether IL-23 gene expression was affected by DSS- or TNBS-induced colitis treatments (**Figure S6e, f**). In DSS-induced colitis, most treatments showed a trend toward decreased IL-23 expression compared with the No therapy group. Notably, *L. lactis* ILP317 in yogurt significantly reduced IL-23 levels, whereas *L. lactis* REX115 did not decrease IL-23 expression and, in fact, led to an increase. In TNBS-induced colitis, unlike the DSS model, *i.p.* administration of ILP317 significantly decreased IL-23 levels, while *i.p.* REX115 showed a non-significant reduction. The other treatments had no marked effect on IL-23 expression.

Together, these cytokine changes, particularly the reduction of IL-6, IL-10, IL-22, and IL-23 in selected treatment groups, support functional attenuation of IL-23-associated inflammatory signalling in the colon after ILP317 and REX115 treatment.

### 3.5. Treatment of DSS colitis mice with *i.p.* REX115 or oral *L. lactis* expressing ILP317 in yoghrt exhibited a trend in the reduction of systemic cytokines

In DSS-induced colitis, the No therapy group showed a significant increase in serum IL-18 and IL-22, and a non-significant increase in serum IL-23 and IL-12p40 (**Figure S7**). A significant decrease in IL-18 was only observed with *i.p.* REX115 protein therapy. No therapy had a significant effect on serum IL-22; a decrease in IL-22 levels was observed only with *i.p.* REX115 and *L. lactis* ILP317 treatments.

In TNBS-induced colitis, no significant changes were detected in the monitored serum immune markers including IL-1β, IL-6, IL-17A, IL-22, IL-23, and TNF-α—and therapy had no significant effect on their levels (Figure S7).

### 3.6. *L. lactis* ILP317 decreases infiltration of neutrophils and eosinophils in the colon of DSS-induced colitis

In a fragment of the large intestine (**Figure S1**) we quantified the immune cell populations using flow cytometry. We analysed CD4^+^ T cells, innate immune cell populations ILC2, ILC3, eosinophils, macrophages, monocytes, and neutrophils (Figure 7a **, Figure S9**). Using tSNE projections, we evaluated cell size, cell number, and marker expression density, with all characteristics visualised in 2-D maps for each population (Figure 7a). The tSNE projection shows a clear decrease of immune cell populations in *L. lactis* ILP317 yogurt treated DSS group in comparison with No therapy DSS group, namely a robust population of neutrophils and eosinophils present in No therapy group was reduced to level of Naïve mice group. A similar effect, but to a lesser extent, was observed in *i.p.* ILP317 group.

**Figure 7.**
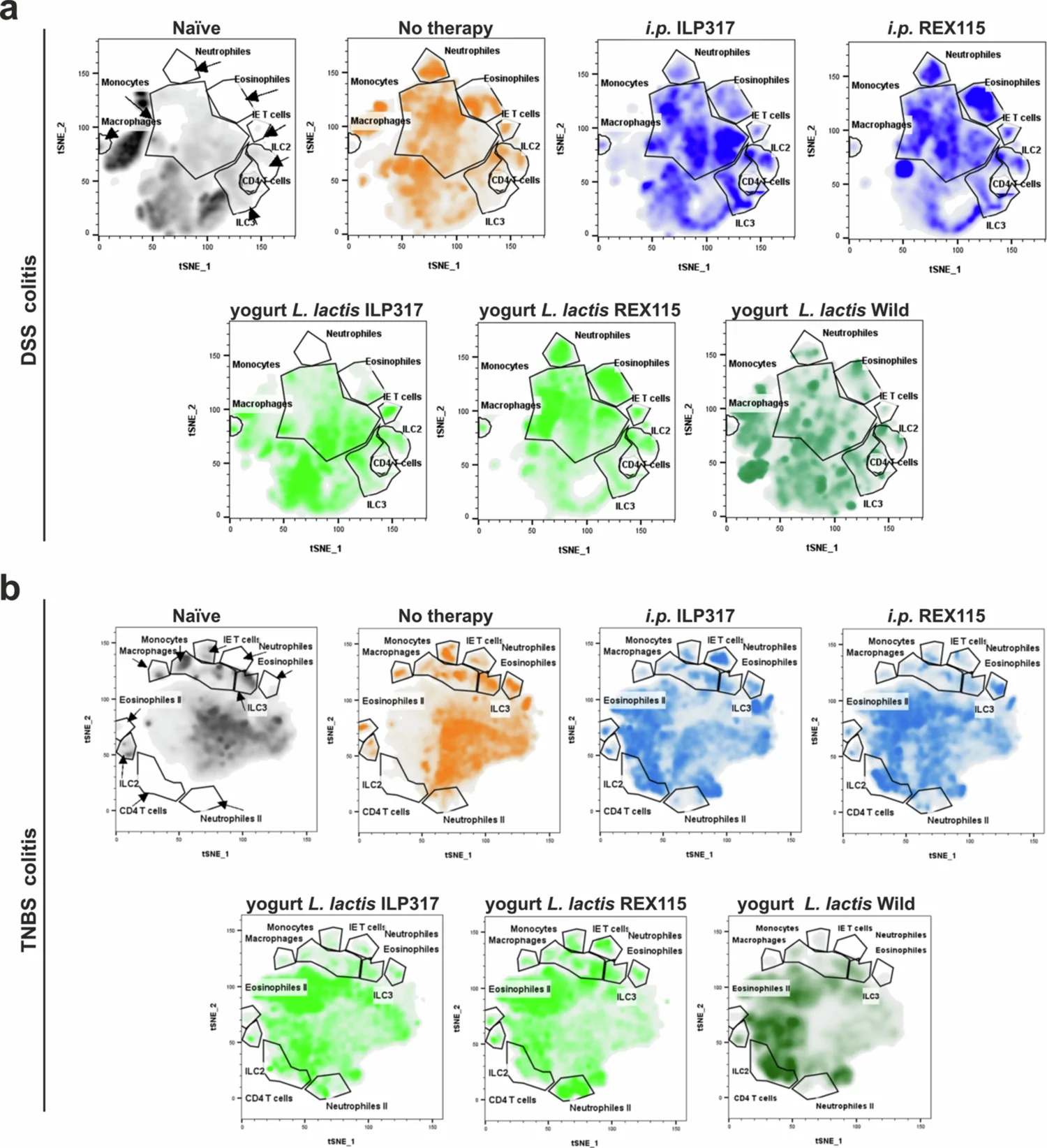
ILP317 and REX115 treatment modulates immune cell populations in the large intestine during experimental colitis. t-SNE projections were used to visualize immune cell populations isolated from the large intestinal mucosa of mice with DSS- or TNBS-induced colitis. **a)** In DSS-induced colitis, DSS treatment was associated with increased accumulation of neutrophils, eosinophils, intraepithelial T cells, and monocytes compared with Naïve mice. Oral administration of *L. lactis* ILP317 produced the clearest shift toward reduced neutrophil and eosinophil density, followed by intraperitoneal ILP317 treatment, whereas the other treatments showed no or only mild effects on immune-cell accumulation in colon tissue. **b)** In TNBS-induced colitis, TNBS treatment was associated mainly with increased neutrophil and eosinophil accumulation compared with Naïve mice. Although *L. lactis* ILP317 showed the most apparent decrease in neutrophil- and eosinophil-associated density patterns, the overall treatment effects on immune-cell populations were milder than in the DSS model. For the DSS experiment, group sizes were as follows: Naïve, *n* = 9; No therapy, *n* = 10; *i.p.* ILP317, *n* = 10; *i.p.* REX115, *n* = 10; *L. lactis* ILP317, *n* = 10; *L. lactis* REX115, *n* = 10; and *L. lactis* Wild, *n* = 10. For the TNBS experiment, group sizes were as follows: Naïve, *n* = 10; No therapy, *n* = 10; *i.p.* ILP317, *n* = 9; *i.p.* REX115, *n* = 9; *L. lactis* ILP317, *n* = 10; *L. lactis* REX115, *n* = 10; *L. lactis* Wild, *n* = 10; gavage *L. lactis* ILP317, *n* = 9; and gavage *L. lactis* REX115, *n* = 9.

TNBS treatment increased presence of neutrophils, eosinophils, monocytes, macrophages and ILC2 populations in comparison with Naïve group of mice. However, we did not observe any decrease in specific immune populations in any of the tested treatments compared to the No therapy TNBS group (Figure 7b).

## 4. Discussion

IBD animal models provide a powerful platform to evaluate causal cytokine-targeted therapies. In clinical practice, parenteral monoclonal antibodies against TNF-*α* and IL-12/IL-23 have proven effective in moderate to severe IBD, improving clinical outcomes, supporting mucosal healing, and prolonging relapse-free periods (Hanauer et al. 2002). In line with these established therapeutic principles, our study demonstrates that selective inhibition of the IL-23/IL-23R axis with recombinant binders (ILP317 and REX115) confers significant protection in experimental colitis. Importantly, oral administration of *L. lactis* engineered to express these binders represents a convenient and non-invasive alternative to systemic therapies, with expected minimal unwanted systemic reactions and evidence of local therapeutic efficacy.

The safety of *L. lactis* as a delivery vehicle has been previously demonstrated in murine IBD models and in a phase I clinical trial (Braat et al. 2006). In particular, genetic replacement of the thymidylate synthase gene with an IL-10 construct generated a transgenic *L. lactis* strain that circumvents safety concerns associated with plasmid-based genetic modification, including the potential dissemination of antibiotic resistance genes, since this engineered *L. lactis* strain cannot survive in the environment in the absence of thymidine (Braat et al. 2006). Thus, the development of strategies for IBD treatment using genetically modified *L. lactis* is promising. Furthermore, systemic side effects associated with conventional administration are avoided (Zadravec et al. 2015; Berlec et al. 2017; Plavec et al. 2019, 2023).

Mechanistically, the protective effect of this delivery strategy is supported by the cytokine and immune-cell changes observed in our experimental models. In the context of DSS- and TNBS-induced epithelial injury, the observed reduction in IL-6, IL-10, and IL-22 expression after ILP317 and REX115 treatment may reflect attenuation of the inflammatory cascade initiated by barrier disruption and DAMP-driven mucosal immune activation. The decrease in IL-6 suggests reduced inflammatory signalling and weaker support for Th17-associated responses (Stockinger and Omenetti 2017), whereas lower IL-22 and IL-10 levels may indicate reduced inflammatory burden and a diminished requirement for compensatory tissue-protective and regulatory responses. Together with reduced neutrophil and eosinophil infiltration, these findings support the interpretation that inhibition of the IL-23/IL-23R axis suppresses both cytokine-mediated and cellular components of intestinal inflammation in these acute colitis models (Siddiqui et al. 2010; Arnold et al. 2016). This interpretation is consistent with the known role of sustained IL-23 signalling in promoting pathogenic Th17 responses, epithelial barrier disruption, and recruitment of inflammatory myeloid cells during intestinal inflammation (Powrie et al. 1993; Siegmund et al. 2001; Uhlig et al. 2006; Yen et al. 2006; Schiering et al. 2014; Kvedaraite et al. 2016).

In this study, using DSS and TNBS colitis models, we compared the effects of IL-23- (ILP317) and IL-23R- (REX115) binding inhibitory proteins administered either via *i.p.* injection of soluble proteins or by oral delivery using yogurt containing recombinant *L. lactis* engineered to express the individual binders (Skrlec et al. 2018; Plavec et al. 2019). This strategy may circumvent degradation of orally administered naked molecules within the gastrointestinal tract (Wang et al. 2016) and could represent a potentially less expensive alternative to currently investigated oral IL-23-targeting therapies, such as the peptide IL-23R blocker Icotrokinra (Mullard 2025). To validate the feasibility of oral administration, we first confirmed that recombinant *L. lactis* remained viable for at least 24 hours after preparation of the yogurt-bacteria mixture. Stool analysis further confirmed the presence of *L. lactis* carrying surface-bound binders in treated animals several hours after administration, consistent with previous reports describing recombinant protein expression by engineered *L. lactis* (Medici et al. 2025). Functionally, our results demonstrate that both ILP317 and REX115 exert significant anti-inflammatory effects; however, the magnitude and range of these effects differed depending on both the colitis model and the route of administration. Interestingly, several differences between intraperitoneal and oral delivery were observed. Intraperitoneal administration ensures systemic bioavailability of soluble binders and likely enables rapid access to mucosal immune compartments. In contrast, oral administration using recombinant *L. lactis* may preferentially provide localised delivery at the intestinal mucosal surface, from where the binders must reach the subepithelial compartment, where IL-23-driven inflammatory responses are initiated and maintained. Local production of inhibitory binders directly within the gastrointestinal tract may therefore represent an important advantage by reducing systemic exposure; however, its efficacy may differ between binders targeting IL-23p19 and those targeting IL-23R.

Although this was not addressed experimentally, one possible explanation is that IL-23R-targeting binders may be partially retained at the epithelial surface through interaction with epithelial IL-23R. Although epithelial IL-23R expression is lower or more context-dependent than that observed in immune cells, such interaction could reduce the availability of REX115 for deeper mucosal immune cell populations, including Th17 cells, ILC3s, γδ T cells, MAIT cells, and colonic Tregs (Arnold et al. 2016).

In DSS-induced colitis, treatment with ILP317 or REX115 significantly ameliorated clinical symptoms, particularly after *i.p*. injection of ILP317 or REX115 or after oral delivery with *L. lactis* ILP317, including reduced DAI scores, prevention of rectal bleeding, and attenuation of colon shortening. Histologically, these treatments significantly decreased inflammatory cell infiltration, preserved mucosal architecture, and prevented epithelial damage. Consistently, ILP317 and REX115 reduced colonic transcription of pro-inflammatory cytokines such as IL-6, IL-22, IL-10, and IL-23, with ILP317 demonstrating a more robust effect when delivered orally via *L. lactis*. Notably, *L. lactis* ILP317 also decreased neutrophil and eosinophil infiltration in the colon, highlighting its ability to modulate innate immune cell populations locally. Together, these cytokine and immune cell changes support functional modulation of IL-23-associated inflammatory pathways, as IL-23 signalling is closely linked to pathogenic Th17-associated responses, cytokine-mediated amplification of mucosal inflammation, and recruitment of innate immune cells. These findings indicate that both systemic and local IL-23 pathway blockade can suppress DSS-induced epithelial injury and associated inflammation, in line with previous studies showing that IL-23 is essential for neutrophil- and eosinophil-mediated tissue damage in acute colitis (Griseri et al. 2015).

In TNBS-induced colitis with frequent colon tissue necrosis and severe ulceration, all tested treatment methods produced a highly significant protective effect at the histological level. Both ILP317 and REX115, administered either intraperitoneally or orally via *L. lactis* in yogurt or, as a control of oral administration, by gavage, markedly reduced inflammatory cell infiltration, preserved mucosal architecture, and prevented epithelial damage, ulceration and necrosis. The protective effects on tissue structure highlight the efficacy of tested topical therapy.

A less pronounced therapeutic effect of the IL-23R-binding protein REX115 delivered orally via recombinant *L. lactis* was observed in both DSS- and TNBS-induced colitis models. We hypothesise that this may result from steric limitations associated with the presentation of REX115 on the bacterial surface, potentially restricting efficient interaction with IL-23R expressed on target cells within the intestinal mucosa. In contrast to intraperitoneal administration of soluble REX115 protein, orally delivered REX115 remains anchored to the bacterial surface, which may reduce accessibility to receptor-binding domains in the complex intestinal microenvironment. Although REX115 is attached to peptidoglycan through a C-terminal AcmA anchoring motif and predicted structural models suggest that this configuration does not directly prevent IL-23R interaction (Plavec and Berlec 2020), the length and flexibility of the peptidoglycan-associated linker may nevertheless limit efficient access to the cytokine homology region of IL-23R. Supporting this interpretation, our previous biosensor studies demonstrated that spacer length within the REX115 fusion construct critically influences IL-23R engagement efficiency (Smejkal et al. 2023). These observations suggest that optimisation of linker architecture may further improve the therapeutic efficacy of orally delivered REX115.

Although the present findings are promising, several translational aspects must be considered before clinical application. The use of genetically modified bacteria in humans requires careful evaluation of biosafety, environmental containment, host immune responses, and long-term safety. Previous studies using engineered *L. lactis* strains demonstrated favourable safety profiles and proposed biological containment strategies limiting environmental persistence. However, while *L. lactis* itself has already been evaluated as a delivery platform in preclinical and early clinical studies, recombinant strains expressing IL-23- or IL-23R-targeting binders have not yet been clinically tested and therefore require further pharmacological and safety validation.

The route of administration is another important translational consideration. Systemic delivery of soluble cytokine-targeting binders may increase the risk of off-target immune modulation and unwanted interference with physiological IL-23 signalling outside the intestinal compartment. In contrast, orally administered recombinant *L. lactis* may provide localised production of inhibitory binders directly at the intestinal mucosal surface, thereby reducing systemic exposure while maintaining therapeutic activity at the site of inflammation.

Dose extrapolation also supports the potential feasibility of this approach, although further optimisation will be required. In the present study, mice received approximately 25 μg of inhibitory binder per 20 g of body weight per day, corresponding to approximately 1.25 mg/kg/day. Using standard body surface area normalisation with the FDA-recommended conversion factor for mice (Km = 3) and humans (Km = 37), this corresponds to a human equivalent dose of approximately 0.1 mg/kg/day. For a 75 kg adult, the estimated dose would therefore be approximately 7.5 mg/day. Although this estimated dose appears pharmacologically feasible, further studies will be required to determine the stability, bioavailability, optimal dosing regimen, and persistence of recombinant *L. lactis*-derived binders within the human gastrointestinal tract under clinical conditions.

Finally, regulatory approval of live biotherapeutic products requires extensive characterisation of genetic stability, manufacturing reproducibility, biological containment, and long-term safety. Future studies should therefore focus on long-term efficacy, detailed mechanistic characterisation, and translational applicability in human IBD, including identification of patient populations that may preferentially benefit from IL-23 versus IL-23R targeting approaches.

Together, these findings support a mechanistic model in which DSS- and TNBS-induced epithelial injury promotes IL-23-associated mucosal inflammation, whereas ILP317- and REX115-mediated interference with the IL-23/IL-23R axis attenuates cytokine-mediated and cellular inflammatory responses, resulting in preserved intestinal architecture and reduced clinical severity of experimental colitis (Figure 8).

**Figure 8.**
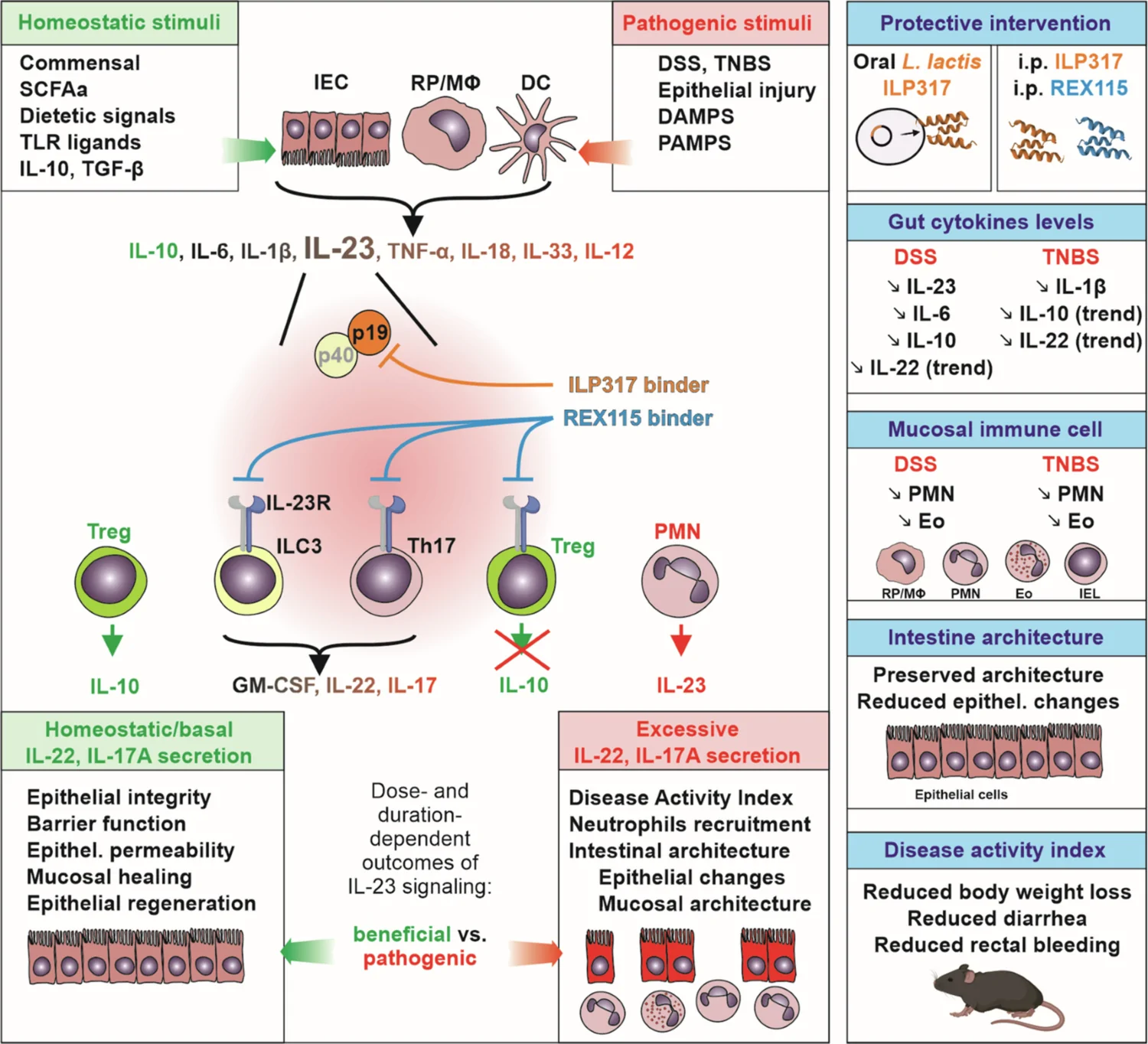
Proposed mechanism of action of IL-23/IL-23R-targeting binders in experimental colitis. The schematic summarizes IL-23/IL-23R signaling in murine colitis and the proposed protective effects of ILP317 and REX115 treatment. Homeostatic microbial stimulation supports basal IL-23-dependent signaling, contributing to controlled IL-22 and IL-17A production, epithelial integrity, and mucosal repair. In contrast, pathogenic stimuli such as DSS- or TNBS-induced epithelial injury activate intestinal epithelial cells, resident phagocytes/macrophages, dendritic cells, and infiltrating inflammatory cells, causes disbiosis leading finally to enhanced production of IL-23 and other pro-inflammatory cytokines. IL-23, composed of p19 and p40 subunits, signals through IL-23R-expressing immune cells, including ILC3, Th17 cells, and Treg cells, thereby promoting GM-CSF, IL-22, and IL-17A production and contributing to recruitment of neutrophils and eosinophils, epithelial damage, and increased disease activity. ILP317 targets the IL-23 cytokine, whereas REX115 targets IL-23R, thereby interfering with IL-23/IL-23R signaling. Protective interventions included intraperitoneal administration of soluble ILP317 or REX115 and oral delivery of recombinant *L. lactis* expressing ILP317 or REX115. In DSS-induced colitis, treatment was associated with reduced colonic expression of IL-23, IL-6, IL-10, and IL-22, as well as decreased neutrophil and eosinophil infiltration. In TNBS-induced colitis, treatment was associated with reduced IL-1β expression and trends toward reduced IL-10 and IL-22 expression. These cytokine and immune-cell changes were accompanied by preserved intestinal architecture, reduced epithelial alterations, and improved clinical parameters, including reduced body weight loss, diarrhea, and rectal bleeding. IEC, intestinal epithelial cells; RP/Mφ, resident phagocytes/macrophages; DC, dendritic cells; ILC3, group 3 innate lymphoid cells; Treg, regulatory T cells; PMN, polymorphonuclear neutrophils; Eo, eosinophils.

In conclusion, this study demonstrates that inhibition of the IL-23/IL-23R axis using ILP317 and REX115 attenuates experimental colitis in murine DSS- and TNBS-induced models. Both soluble binders administered intraperitoneally and recombinant *L. lactis*-based oral delivery showed anti-inflammatory effects, including improvement of clinical and histological parameters and modulation of inflammatory cytokine expression. Among the orally delivered approaches, *L. lactis* expressing ILP317 showed more consistent protective effects, likely reflecting efficient neutralisation of soluble IL-23 within the inflamed intestinal environment.

These findings support recombinant *L. lactis* as a promising preclinical platform for localised mucosal delivery of cytokine-targeting binders. However, the results should be interpreted within the limitations of acute murine colitis models, and further studies are required to assess long-term efficacy, safety, dosing, regulatory feasibility, and translational relevance in human IBD.

## Supplementary Material

Supplementary Material ARRIVE_g_Author_Checklist.pdf

Supplementary Material Supplementary materials_revision_minor.docx

## Data Availability

All data supporting the findings of this study are available within the paper and its Supplementary Information file.

## Data sharing statement

All experimental data not included in the manuscript will be available upon request immediately following publication. No additional datasets were generated or analysed during the current study. Specific material will be available upon materials transfer agreement.

## References

[cit0001] Arnold IC , Mathisen S , Schulthess J et al. 2016. CD11c^+^ monocyte/macrophages promote chronic *helicobacter hepaticus*-induced intestinal inflammation through the production of IL-23. Mucosal Immunol. 9(2):352–363. 10.1038/mi.2015.65 PMC4650208 26242598

[cit0002] Becker C , Dornhoff H , Neufert C et al. 2006. Cutting edge: IL-23 cross-regulates IL-12 production in T cell-dependent experimental colitis. J Immunol. 177(5):2760–2764. 10.4049/jimmunol.177.5.2760 16920909

[cit0003] Berlec A , Strukelj B . 2009. Novel applications of recombinant lactic acid bacteria in therapy and in metabolic engineering. Recent Pat Biotechnol. 3(2):77–87. 10.2174/187220809788700175 19519564

[cit0004] Berlec A , Ravnikar M , Strukelj B . 2012. Lactic acid bacteria as oral delivery systems for biomolecules. Pharmazie. 67(11):891–898. 23210237

[cit0005] Berlec A , Perse M , Ravnikar M et al. 2017. Dextran sulphate sodium colitis in C57BL/6J mice is alleviated by lactococcus lactis and worsened by the neutralization of tumor necrosis factor alpha. Int Immunopharmacol. 43:219–226. 10.1016/j.intimp.2016.12.027 28039805

[cit0006] Braat H , Rottiers P , Hommes DW et al. 2006. A phase I trial with transgenic bacteria expressing interleukin-10 in Crohn's disease. Clin Gastroenterol Hepatol. 4(6):754–759. 10.1016/j.cgh.2006.03.028 16716759

[cit0007] Cox JH , Kljavin NM , Ota N et al. 2012. Opposing consequences of IL-23 signaling mediated by innate and adaptive cells in chemically induced colitis in mice. Mucosal Immunol. 5(1):99–109. 10.1038/mi.2011.54 22089030

[cit0008] Duerr RH , Taylor KD , Brant SR et al. 2006. A genome-wide association study identifies *il23r* as an inflammatory bowel disease gene. Sci. 314(5804):1461–1463. 10.1126/science.1135245 PMC4410764 17068223

[cit0009] Elson CO , Cong Y , Weaver CT et al. 2007. Monoclonal anti-interleukin 23 reverses active colitis in a T cell-mediated model in mice. Gastroenterology. 132(7):2359–2370. 10.1053/j.gastro.2007.03.104 17570211

[cit0010] Erben U , Loddenkemper C , Doerfel K et al. 2014. A guide to histomorphological evaluation of intestinal inflammation in mouse models. Int J Clin Exp Pathol. 7(8):4557–4576. PMC4152019 25197329

[cit0011] Feagan BG , Sandborn WJ , D'Haens G et al. 2017. Induction therapy with the selective interleukin-23 inhibitor risankizumab in patients with moderate-to-severe Crohn's disease: a randomised, double-blind, placebo-controlled phase 2 study. Lancet. 389(10080):1699–1709. 10.1016/S0140-6736(17)30570-6 28411872

[cit0012] Friedrich M , Pohin M , Powrie F . 2019. Cytokine networks in the pathophysiology of inflammatory bowel disease. Immunity. 50(4):992–1006. 10.1016/j.immuni.2019.03.017 30995511

[cit0013] Fung TC , Bessman NJ , Hepworth MR et al. 2016. Lymphoid-tissue-resident commensal bacteria promote members of the IL-10 cytokine family to establish mutualism. Immunity. 44(3):634–646. 10.1016/j.immuni.2016.02.019 PMC4845739 26982365

[cit0014] Geremia A , Jewell DP . 2012. The IL-23/IL-17 pathway in inflammatory bowel disease. Expert Rev Gastroenterol Hepatol. 6(2):223–237. 10.1586/egh.11.107 22375527

[cit0015] Griseri T , Arnold IC , Pearson C et al. 2015. Granulocyte macrophage colony-stimulating factor-activated eosinophils promote Interleukin-23 driven chronic colitis. Immunity. 43(1):187–199. 10.1016/j.immuni.2015.07.008 PMC4518500 26200014

[cit0016] Groza Y , Lacina L , Kuchař M et al. 2024. Small protein blockers of human IL-6 receptor alpha inhibit proliferation and migration of cancer cells. Cell Commun Signal. 22(1):261. 10.1186/s12964-024-01630-w PMC11075285 38715108

[cit0017] Guan Q , Weiss CR , Wang S et al. 2018. Reversing ongoing chronic intestinal inflammation and fibrosis by sustained block of IL-12 and IL-23 using a vaccine in mice. Inflamm Bowel Dis. 24(9):1941–1952. 10.1093/ibd/izy142 PMC6205364 29788053

[cit0018] Hanauer SB , Feagan BG , Lichtenstein GR et al. 2002. Maintenance infliximab for Crohn's disease: the ACCENT I randomised trial. Lancet. 359(9317):1541–1549. 10.1016/S0140-6736(02)08512-4 12047962

[cit0019] Hunter CA , Jones SA . 2015. IL-6 as a keystone cytokine in health and disease. Nat Immunol. 16(5):448–457. 10.1038/ni.3153 25898198

[cit0020] Ivanov II , Atarashi K , Manel N et al. 2009. Induction of intestinal Th17 cells by segmented filamentous bacteria. Cell. 139(3):485–498. 10.1016/j.cell.2009.09.033 PMC2796826 19836068

[cit0021] Juritsch AF , Moreau R . 2019. Rapid removal of dextran sulfate sodium from tissue RNA preparations for measurement of inflammation biomarkers. Anal Biochem. 579:18–24. 10.1016/j.ab.2019.05.011 31112717

[cit0022] Kontoyiannis D , Pasparakis M , Pizarro TT et al. 1999. Impaired on/off regulation of TNF biosynthesis in mice lacking TNF AU-rich elements: implications for joint and gut-associated immunopathologies. Immunity. 10(3):387–398. 10.1016/s1074-7613(00)80038-2 10204494

[cit0023] Krizova L , Kuchar M , Petrokova H et al. 2017. p19-targeted ABD-derived protein variants inhibit IL-23 binding and exert suppressive control over IL-23-stimulated expansion of primary human IL-17+ T-cells. Autoimmunity. 50(2):102–113. 10.1080/08916934.2016.1272598 28100093

[cit0024] Kuchar M , Vankova L , Petrokova H et al. 2014. Human interleukin-23 receptor antagonists derived from an albumin-binding domain scaffold inhibit IL-23-dependent ex vivo expansion of IL-17-producing T-cells. Proteins. 82(6):975–989. 10.1002/prot.24472 PMC4285857 24549990

[cit0025] Kuchař M , Sloupenská K , Rašková Kafková L et al. 2024. Human IL-22 receptor-targeted small protein antagonist suppress murine DSS-induced colitis. Cell Commun Signal. 22(1):469. 10.1186/s12964-024-01846-w PMC11446014 39354587

[cit0026] Kullberg MC , Jankovic D , Feng CG et al. 2006. IL-23 plays a key role in helicobacter hepaticus-induced T cell-dependent colitis. J Ex Med. 203(11):2485–2494. 10.1084/jem.20061082 PMC2118119 17030948

[cit0027] Kvedaraite E , Lourda M , Idestrom M et al. 2016. Tissue-infiltrating neutrophils represent the main source of IL-23 in the colon of patients with IBD. Gut. 65(10):1632–1641. 10.1136/gutjnl-2014-309014 26160381

[cit0028] Livak KJ , Schmittgen TD . 2001. Analysis of relative gene expression data using real-time quantitative PCR and the 2(-Delta Delta C(T)) Method. Methods. 25(4):402–408. 10.1006/meth.2001.1262 11846609

[cit0029] Macho-Fernandez E . 2015. Lymphotoxin beta receptor signaling limits mucosal damage through driving IL-23 production by epithelial cells. Mucosal Immunol. 8. 10.1038/mi.2014.78 PMC4364000 25183367

[cit0030] McDonald BD , Dyer EC , Rubin DT . 2022. IL-23 monoclonal antibodies for IBD: so many, so different? J Crohns Colitis. 16(Supplement_2):ii42–ii53. 10.1093/ecco-jcc/jjac038 PMC9097671 35553664

[cit0031] Medici G , Candini G , Mottolese N et al. 2025. Lactic acid bacteria as microbial cell factories for the in vivo delivery of therapeutic proteins as secretable TAT fusion products. J Biol Eng. 19(1):65. 10.1186/s13036-025-00538-4 PMC12273004 40676698

[cit0032] Morris GP , Beck PL , Herridge MS et al. 1989. Hapten-induced model of chronic inflammation and ulceration in the rat colon. Gastroenterology. 96(3):795–803. 2914642

[cit0033] Mullard A . 2025. Oral IL-23-blocking peptide racks up phase III wins in inflammatory disease. Nat Rev Drug Discov. 24(5):321. 10.1038/d41573-025-00063-5 40169875

[cit0034] Okayasu I , Hatakeyama S , Yamada M et al. 1990. A novel method in the induction of reliable experimental acute and chronic ulcerative colitis in mice. Gastroenterology. 98(3):694–702. 10.1016/0016-5085(90)90290-h 1688816

[cit0035] Oppmann B , Lesley R , Blom B et al. 2000. Novel p19 protein engages IL-12p40 to form a cytokine, IL-23, with biological activities similar as well as distinct from IL-12. Immunity. 13(5):715–725. 10.1016/s1074-7613(00)00070-4 11114383

[cit0036] Plavec TV , Berlec A . 2020. Safety aspects of genetically modified lactic acid bacteria. Microorganisms. 8(2). 10.3390/microorganisms8020297 PMC7074969 32098042

[cit0037] Plavec TV , Klemencic K , Kuchar M et al. 2023. Secretion and surface display of binders of IL-23/IL-17 cytokines and their receptors in lactococcus lactis as a therapeutic approach against inflammation. Eur J Pharm Sci. 190:106568. 10.1016/j.ejps.2023.106568 37619953

[cit0038] Plavec TV , Kuchar M , Benko A et al. 2019. Engineered lactococcus lactis secreting IL-23 receptor-targeted REX protein blockers for modulation of IL-23/Th17-Mediated inflammation. Microorganisms. 7(5). 10.3390/microorganisms7050152 PMC6560508 31137908

[cit0039] Powrie F , Leach MW , Mauze S et al. 1993. Phenotypically distinct subsets of CD4(+) T-cells induce or protect from chronic intestinal inflammation in C-B-17 SCID mice. Int Immunol. 5(11):1461–1471. 10.1093/intimm/5.11.1461 7903159

[cit0040] Sands BE , Chen JJ , Feagan BG et al. 2017. Efficacy and safety of MEDI2070, an antibody against interleukin 23, in patients with moderate to severe Crohn's disease: a phase 2a study. Gastroenterology. 153(1):77. 10.1053/j.gastro.2017.03.049 28390867

[cit0041] Schiering C , Krausgruber T , Chomka A et al. 2014. The alarmin IL-33 promotes regulatory T-cell function in the intestine. Natur. 513(7519):564–568. 10.1038/nature13577 PMC4339042 25043027

[cit0042] Sedhom MAK , Pichery M , Murdoch JR et al. 2013. Neutralisation of the interleukin-33/ST2 pathway ameliorates experimental colitis through enhancement of mucosal healing in mice. Gut. 62(12):1714–1723. 10.1136/gutjnl-2011-301785 PMC3841767 23172891

[cit0043] Sewell GW , Kaser A . 2022. Interleukin-23 in the pathogenesis of inflammatory bowel disease and implications for therapeutic intervention. J Crohns Colitis. 16(Supplement_2):ii3–ii19. 10.1093/ecco-jcc/jjac034 PMC9097674 35553667

[cit0044] Siddiqui KRR , Laffont S , Powrie F . 2010. E-Cadherin marks a subset of inflammatory dendritic cells that promote T cell-mediated colitis. Immunity. 32(4):557–567. 10.1016/j.immuni.2010.03.017 PMC2938478 20399121

[cit0045] Siegmund B , Lehr HA , Fantuzzi G et al. 2001. IL-1β-converting enzyme (caspase-1) in intestinal inflammation. Proc Natl Acad Sci USA. 98(23):13249–13254. 10.1073/pnas.231473998 PMC60856 11606779

[cit0046] Siegmund B , Fantuzzi G , Rieder F et al. 2001. Neutralization of interleukin-18 reduces severity in murine colitis and intestinal IFN-γ and TNF-α production. Am J Physiol-Regul Integr Comp Physiol.. 281(4):R1264–R73. 10.1152/ajpregu.2001.281.4.R1264 11557635

[cit0047] Skrlec K , Zadravec P , Hlavnickova M et al. 2018. p19-Targeting ILP protein blockers of IL-23/Th-17 pro-inflammatory axis displayed on engineered bacteria of food origin. Int J Mol Sci. 19(7). 10.3390/ijms19071933 PMC6073689 29966384

[cit0048] Smejkal J , Aubrecht P , Semeradtova A et al. 2023. Immunocapturing rare cells from blood: a simple and robust microsystem approach. Biosens Bioelectron. 227:115155. 10.1016/j.bios.2023.115155 36821992

[cit0049] Stockinger B , Omenetti S . 2017. The dichotomous nature of T helper 17 cells. Nat Rev Immunol. 17(9):535–544. 10.1038/nri.2017.50 28555673

[cit0050] Uhlig HH , McKenzie BS , Hue S et al. 2006. Differential activity of IL-12 and IL-23 in mucosal and systemic innate immune pathology. Immunity. 25(2):309–318. 10.1016/j.immuni.2006.05.017 16919486

[cit0051] Van Limbergen J , Wilson DC , Satsangi J . 2009. The genetics of Crohn's disease. Annu Rev Genomics Hum Genet. 89–116. 10.1146/annurev-genom-082908-150013 19453248

[cit0052] Verstockt B , Salas A , Sands BE et al. 2023. IL-12 and IL-23 pathway inhibition in inflammatory bowel disease. Nat Rev Gastroenterol Hepatol. 20(7):433–446. 10.1038/s41575-023-00768-1 PMC10958371 37069321

[cit0053] Wang M , Gao Z , Zhang Y et al. 2016. Lactic acid bacteria as mucosal delivery vehicles: a realistic therapeutic option. Appl Microbiol Biotechnol. 100(13):5691–5701. 10.1007/s00253-016-7557-x 27154346

[cit0054] Wang H , Chao K , Ng SC et al. 2016. Pro-inflammatory miR-223 mediates the cross-talk between the IL23 pathway and the intestinal barrier in inflammatory bowel disease. Genome Biol. 17:58. 10.1186/s13059-016-0901-8 PMC4815271 27029486

[cit0055] Xavier RJ , Podolsky DK . 2007. Unravelling the pathogenesis of inflammatory bowel disease. Natur. 448(7152):427–434. 10.1038/nature06005 17653185

[cit0056] Yen D , Cheung J , Scheerens H et al. 2006. IL-23 is essential for T cell-mediated colitis and promotes inflammation via IL-17 and IL-6. J Clin Invest. 116(5):1310–1316. 10.1172/JCI21404 PMC1451201 16670770

[cit0057] Zadravec P , Strukelj B , Berlec A . 2015. Heterologous surface display on lactic acid bacteria: non-GMO alternative? Bioengineered. 6(3):179–183. 10.1080/21655979.2015.1040956 PMC4601214 25880164

