# Supplementary material for: Oral delivery of recombinant Lactococcus lactis expressing an IL-23 inhibitor as a potential therapeutic strategy for experimental colitis
Source: Drug Deliv. 2026 Oct 3;33(1):2726654. doi: 10.1080/10717544.2026.2726654 (PMC13644427; doi:10.1080/10717544.2026.2726654)
Supplement: Supplementary file 1 [file IDRD_A_2726654_SM2473.pdf]

NOTE: Please save this file locally before filling in the table, DO NOT work on the file within your internet browser as changes will not be saved. Adobe Acrobat Reader (available free here) is recommended for completion.

The ARRIVE guidelines 2.0: author checklist

The ARRIVE Essential 10

These items are the basic minimum to include in a manuscript. Without this information, readers and reviewers cannot assess the reliability of the findings.

Item		Recommendation	Section/line number, or reason for not reporting
**Study design**	1	For each experiment, provide brief details of study design including:	
		a. The groups being compared, including control groups. If no control group has been used, the rationale should be stated.	Supplementary material: Table S2. Experimental groups of C57BL/6 mice in DSS experiments, Table S3. Experimental groups of BALBc mice in TNBS experiments.
		b. The experimental unit (e.g. a single animal, litter, or cage of animals).	cage of animals
**Sample size**	2	a. Specify the exact number of experimental units allocated to each group, and the total number in each experiment. Also indicate the total number of animals used.	Table S2 and Table S3 in Supplementary Materials
		b. Explain how the sample size was decided. Provide details of any *a priori* sample size calculation, if done.	The sample size was set to n = 10 mice per group based on (i) common practice in comparable preclinical mouse studies, (ii) feasibility considerations (including animal housing capacity, time constraints, and
**Inclusion and exclusion criteria**	3	a. Describe any criteria used for including and excluding animals (or experimental units) during the experiment, and data points during the analysis. Specify if these criteria were established *a priori*. If no criteria were set, state this explicitly.	Inclusion and exclusion criteria were defined a priori before the start of the experiment. Animals were included if they met the predefined eligibility requirements at baseline (e.g., [strain/sex/age/weight range; health
		b. For each experimental group, report any animals, experimental units or data points not included in the analysis and explain why. If there were no exclusions, state so.	all animals were included
		c. For each analysis, report the exact value of *n* in each experimental group.	all animals were included
**Randomisation**	4	a. State whether randomisation was used to allocate experimental units to control and treatment groups. If done, provide the method used to generate the randomisation sequence.	Animals were housed in an animal facility at Palacky University, Faculty of Medicine and Dentistry in accordance with ARRIVE and Institutional Animal Care guidelines. All experimental protocols were approved by the Ethics Committee of the Faculty of Medicine and Dentistry (Palacky University Olomouc,
		b. Describe the strategy used to minimise potential confounders such as the order of treatments and measurements, or animal/cage location. If confounders were not controlled, state this explicitly.	Potential confounding factors were minimised through experimental design and procedural controls. The order of treatments and measurements was randomised using a predefined randomisation scheme to avoid systematic order effects. Animals were randomly allocated to experimental groups
**Blinding**	5	Describe who was aware of the group allocation at the different stages of the experiment (during the allocation, the conduct of the experiment, the outcome assessment, and the data analysis).	Group allocation was performed by a researcher not involved in the conduct of the experiment. During the experimental procedures, the personnel responsible for animal handling and treatment administration were aware of group allocation due to the nature of the interventions. Outcome
**Outcome measures**	6	a. Clearly define all outcome measures assessed (e.g. cell death, molecular markers, or behavioural changes).	DAI, Gene expression of selected cytokines in the gut, cytokine expression in the serum, colon lenght, histomorphology assesment
		b. For hypothesis-testing studies, specify the primary outcome measure, i.e. the outcome measure that was used to determine the sample size.	For this hypothesis-testing study, the primary outcome measure was DAI, colon lenght, colon tissue histology, cytokine expression in colon tissue and in the blood. This outcome was defined a priori and was used as the
**Statistical methods**	7	a. Provide details of the statistical methods used for each analysis, including software used.	Statistical analyses were performed using [GraphPad Prism 8]. Data were first assessed for distributional characteristics using the Shapiro – Wilk test due to the small sample size (n = 10). As the assumptions for
		b. Describe any methods used to assess whether the data met the assumptions of the statistical approach, and what was done if the assumptions were not met.	Prior to statistical analysis, data were assessed for compliance with the assumptions of the selected statistical approach. Normality of data distribution was evaluated using the Shapiro – Wilk test,
**Experimental animals**	8	a. Provide species-appropriate details of the animals used, including species, strain and substrain, sex, age or developmental stage, and, if relevant, weight.	Mice were of the BALB/C and C57BL/6 strain, females and 8-9 weeks old at the start of the experiment.
		b. Provide further relevant information on the provenance of animals, health/immune status, genetic modification status, genotype, and any previous procedures.	All animals used in this study were laboratory mice obtained from [Anlab s.r.o., Czech Republic]. Health and immune status were monitored regularly by the animal facility, and all animals were free of clinically relevant
**Experimental procedures**	9	For each experimental group, including controls, describe the procedures in enough detail to allow others to replicate them, including:	
		a. What was done, how it was done and what was used.	Acute colitis was induced in C57BL/6 mice by oral administration of 2.5% (w/v) DSS (MW approximately 40 kDa; TdB Labs, Uppsala, Sweden) in drinking water for 4
		b. When and how often.	Preparation of food for one mouse: 2×109 CFU of recombinant or wild type L. lactis were mixed with 3 g of white yogurt (2.7% fat; OLMA, Olomouc, Czech Republic)
		c. Where (including detail of any acclimatisation periods).	8-9 weeks old female C57BL/6 or BALB/c mice (AnLab, Prague, Czech Republic) weighing between 18 and 22 g before treatment were kept under standardized
		d. Why (provide rationale for procedures).	Standardised procedures were used to induce chronic and acute colitis.
**Results**	10	For each experiment conducted, including independent replications, report:	
		a. Summary/descriptive statistics for each experimental group, with a measure of variability where applicable (e.g. mean and SD, or median and range).	all results are present in enclosed manuscript
		b. If applicable, the effect size with a confidence interval.	all results are present in enclosed manuscript
