# Supplementary material for: Oral delivery of recombinant Lactococcus lactis expressing an IL-23 inhibitor as a potential therapeutic strategy for experimental colitis
Source: Drug Deliv. 2026 Oct 3;33(1):2726654. doi: 10.1080/10717544.2026.2726654 (PMC13644427; doi:10.1080/10717544.2026.2726654)
Supplement: Supplementary file 2 [file IDRD_A_2726654_SM3086.docx]

**Supplementary materials**

**Oral delivery of recombinant *Lactococcus lactis* expressing an IL-23 inhibitor as a potential therapeutic strategy for experimental colitis**

Eliska Kopecna ^a,1^, Leona Raskova Kafkova ^a,1,^*, Petr Kosztyu ^a^, Kristyna Sloupenska ^a^, Katerina Zachova ^a^, Alica Cutkova ^a^, Jozef Skarda ^b,c^, Jana Vaculova ^b^, Milan Kuchar ^d^, Natalya Panova ^d^, Stephen Walimbwa ^a^, Tina Vida Plavec ^e,f^, Ales Berlec ^e,f^, Petr Maly ^d,^*, and Milan Raska ^a,^*

**Table S1. List of primers used in our study.**

| **Gene** | **Primer** | **Sequence (5´ to 3´)** |
| --- | --- | --- |
| **GAPDH** | Forward | CGTCCCGTAGACAAAATGGT |
|  | Reverse | TCAATGAAGGGGTCGTTGAT |
| **IL-1ß** | Forward | TGCCACCTTTTGACAGTGATG |
|  | Reverse | ATGTGCTGCTGCGAGATTTG |
| **TNF-α** | Forward | AAGCCTGTAGCCCACGTCGTA |
|  | Reverse | AGGTACAACCCATCGGCTGG |
| **IL-6** | Forward | ACAAAGCCAGAGTCCTTCAGAG |
|  | Reverse | GCCACTCCTTCTGTGACTCC |
| **IL-10** | Forward | TGAATTCCCTGGGTGAGAAG |
|  | Reverse | TGGCCTTGTAGACACCTTGG |
| **IL-22** | Forward | GGTGACGACCAGAACATCCA |
|  | Reverse | GACGTTAGCTTCTCACTTTCCTT |
| **IL-12** | Forward | ATTACTCCGGACGGTTCACG |
|  | Reverse | ACGCCATTCCACATGTCACT |
| **IL-17A** | Forward | TTTTCAGCAAGGAATGTGGA |
|  | Reverse | TTCATTGTGGAGGGCAGAC |
| **IL-22BP** | Forward | TCAGCAGCAAAGACAGAAGAAAC |
|  | Reverse | GTGTCTCCAGCCCAACTCTCA |
| **IL-23** | Forward | AACAGCTTAAGGATGCCCAGGTTC |
|  | Reverse | ATAATGGTGTCCTTGCCCTTCACG |

**Table S2.** **Experimental groups of C57BL/6 mice in DSS experiments.**

Experimental group 1

| Group designation | No. of animals | DSS | Route of ILP/REX administration |
| --- | --- | --- | --- |
| Naïve | 5 | no | none |
| “No therapy” | 5 | yes | none |
| *i.p.* ILP317 | 10 | yes | *i.p.* |
| *i.p.* REX115 | 10 | yes | *i.p.* |
| *L. lactis* ILP317 | 10 | yes | oral |
| *L. lactis* REX115 | 10 | yes | oral |
| *L. lactis* “Wild” | 5 | yes | oral |

Experimental group 2

| Group designation | No. of animals | DSS | Route of ILP/REX administration |
| --- | --- | --- | --- |
| Naïve | 9 | no | none |
| “No therapy” | 10 | yes | none |
| *i.p.* ILP317 | 10 | yes | *i.p.* |
| *i.p.* REX115 | 10 | yes | *i.p.* |
| *L. lactis* ILP317 | 10 | yes | oral |
| *L. lactis* REX115 | 10 | yes | oral |
| *L. lactis* “Wild” | 10 | yes | oral |

**Table S3. Experimental groups of BALBc mice in TNBS experiments.**

Experimental group 1

| Group designation | No. of animals | TNBS | Route of ILP/REX  administration |
| --- | --- | --- | --- |
| Naïve | 10 | no | none |
| “No therapy” | 10 | yes | none |
| *i.p.* ILP317 | 9 | yes | *i.p.* |
| *i.p.* REX115 | 9 | yes | *i.p.* |
| *L. lactis* ILP317 | 10 | yes | oral |
| *L. lactis* REX115 | 10 | yes | oral |
| *L. lactis* “Wild” | 10 | yes | oral |
| Gav ILP317 | 9 | yes | gavage |
| Gav REX115 | 9 | yes | gavage |

Experimental group 2

| Group designation | No. of animals | TNBS | Route of ILP/REX  administration |
| --- | --- | --- | --- |
| Naïve | 10 | no | none |
| “No therapy” | 12 | yes | none |
| *L. lactis* ILP317 | 10 | yes | oral |
| *L. lactis* “Wild” | 5 | yes | oral |

**Supplementary methods**

**Sample size justification and power analysis**

Sample sizes were determined based on previous experience with DSS- and TNBS-induced colitis models, expected inter-individual variability in clinical, macroscopic, histological, and inflammatory parameters, and ethical principles aimed at reducing animal use. For the main therapeutic intervention groups, 9–10 animals per group were used. Power analysis indicated that, for a two-sided comparison with α = 0.05 and 80% power, n = 10 animals per group is sufficient to detect a standardized effect size of approximately Cohen’s d = 1.32, while n = 9 animals per group allows detection of an effect size of approximately Cohen’s d = 1.41. These values correspond to large treatment effects, which are expected in acute DSS- and TNBS-induced colitis models when comparing untreated colitis mice with mice receiving effective anti-inflammatory treatment.

Naïve and selected control groups containing 5 animals were included primarily as reference groups to verify baseline parameters or disease induction and were not considered the principal powered efficacy comparisons. The main treatment comparisons were performed between colitis untreated animals and the corresponding ILP317-, REX115-, or recombinant *L. lactis*-treated groups.

**Measurement of binding kinetics using LigandTracer Green Line**

NIH-3T3 and COS-7 cells were cultured in DMEM supplemented with 10% fetal calf serum, glutamine, and antibiotic–antimycotic solution (penicillin/streptomycin) at 37 °C in a humidified atmosphere containing 5% CO₂. Cells were detached by scraping and counted using a hemocytometer. Approximately 1 × 10⁶ cells in 2 mL of growth medium were seeded onto a tilted 87 mm cell culture dish (Nunclon™, Cat. No. 150350). The dish was kept in the tilted position and incubated overnight. The following day, the medium was carefully removed, the dish was placed horizontally, and 10 mL of fresh growth medium was added. Cells were then incubated overnight under standard culture conditions.

Dialyzed in vivo biotinylated His6-REX115-TolA-AviTag protein was diluted in measurement medium to final concentrations of 3, 10, and 30 nM in a total volume of 3 mL. Streptavidin-APC was then added to a final concentration of 625 nM, and the mixture was incubated for 30 min at room temperature. Binding kinetics were measured sequentially by adding increasing ligand concentrations to the cells. After incubation with the first ligand concentration, the measurement was paused and the ligand-containing mixture was added to the cell culture dish. The same procedure was repeated for the second and third ligand concentrations. Once the binding signal reached saturation, the measurement was paused, the ligand-containing medium was removed, and fresh ligand-free medium was added. Dissociation was then recorded until more than 20% of the ligand had dissociated from the cells. Data were analyzed using TraceDrawer software (Ridgeview Instruments AB, Uppsala, Sweden).

**ELISA-based binding assay for ILP317 interaction with murine IL-23**

Murine IL-23-Fc-IgG protein was immobilized on a PolySorp 96-well immunoplate at a concentration of 5 µg/mL. After blocking with 1% BSA in PBS containing 0.01% Tween, in vivo biotinylated ILP317 (His₆-ILP317-TolA-Avi) was added as a serially diluted analyte. Binding of ILP317 to murine IL-23-coated wells or BSA-blocked control wells was detected using HRP-conjugated streptavidin diluted 1:10,000.

**Figure S1.** **Division of the colon into samples for individual analyses.**

The first part from rectal side was used for immunohistochemistry (IHC), the second for qRT-PCR, the third for flow cytometry and the fourth for qRT-PCR. Two parts for qRT-PCR were pooled and analyzed together. This order was strictly followed in all colitis experiments.

**Figure S2. Binding of ABD-derived protein blockers to murine IL-23 cytokine and its cognate murine receptor. A)** **Binding of in vivo biotinylated ILP317 (His₆-ILP317-TolA-Avi) to immobilized murine IL-23-Fc-IgG was assessed by ELISA.** ILP317 showed concentration-dependent binding to murine IL-23, whereas only minimal signal was detected in BSA-blocked control wells, confirming specific interaction with murine IL-23. Data are presented as mean ± SD of triplicate wells.
**B)** **Real-time binding kinetics of in vivo biotinylated REX115 (His₆-REX115-TolA-Avi) to NIH-3T3 and COS-7 cells were measured using LigandTracer Green Line.** REX115 showed high-affinity binding to NIH-3T3 cells, representing murine IL-23R-expressing cells, with an estimated Kd of 0.1 nM. Binding to COS-7 cells was also detected; however, the binding signal was lower compared with NIH-3T3 cells and the estimated Kd was 1.0 nM.

Black and red curves represent measured binding signals, while fitted curves indicate kinetic model fitting.

**Figure S3. Analysis of viability of *L. lactis* producing REX115 or ILP317 was not affected by storage at 4°C or 22°C for 24 h.**

Induced and non-induced *L. lactis* cells expressing ILP317 or REX115 binders, mixed with yogurt, were incubated at 4 °C or 22 °C for 12 and 24 hours. Bacterial viability was assessed by plating on selective GM17 agar containing chloramphenicol and quantified as CFU.

**Figure S4. Viability check of *L. lactis* isolated from pellets.**

A total of 2 × 10⁹ *L. lactis* cells were either dispersed in yogurt and applied to standard chow, which was then provided to mice for 1 or 24 hours, or administered directly by gavage. Fecal pellets were collected at the indicated time points, and *L. lactis* was isolated and quantified as CFU on selective GM17 agar plates containing chloramphenicol.

For this experiment Naïve group of 5 mice were used.

**Figure S5. Representative examples of colon dissected from mice with a) DSS- and b) TNBS-induced colitis.**

**Figure S6. Gene expression of cytokines in DSS- and TNBS-induced colitis.**

qRT-PCR analysis of **a)** IL-17A, **b)** TNF-α, and **c)** IL-22BP, **d)** IL-1β, **e)** IL-23 expression in DSS-induced colitis and **f)** IL-23 expression in TNBS-induced colitis. Statistical differences were analyzed by Kruskal-Wallis one-way ANOVA followed by Dunn´s multiple comparisons test. Statistical significance is shown relative to the No therapy group. Means +/- SD are shown. * P<0.05, ** P<0.01, *** P<0.001.

For the DSS experiment, group sizes were as follows: *L. lactis* ILP317, *n* = 10; *L. lactis* REX115, *n* = 10. For the TNBS experiment, group sizes were as follows: *L. lactis* ILP317, *n* = 10; *L. lactis* REX115, *n* = 10; gavage *L. lactis* ILP317, *n* = 9; and gavage *L. lactis* REX115, *n* = 9.

**Figure S7. Serum cytokines were not affected by any REX115 and ILP317 therapy used to treat DSS colitis.**

Serum levels of IL-17A, IL-18, IL-22, IL-23, and its subunit IL-12p40 were measured by flow cytometry using fluorescent microbeads conjugated with an antibody targeting particular cytokine.

For the DSS experiment, group sizes were as follows: *L. lactis* ILP317, *n* = 10; *L. lactis* REX115, *n* = 10.

Statistical significance evaluated by Kruskal-Wallis one-way ANOVA model followed by Dunn´s multiple comparisons test is shown relative to No therapy group of mice. Means +/- SD are shown. ** P<0.01.

**Figure S8. Serum cytokines were not affected by any REX115 and ILP317 therapy used to treat TNBS colitis.**

Serum levels of IL-1β, IL-6, IL-17A, IL-22, IL-23, and TNF-α were measured by flow cytometry using fluorescent microbeads conjugated with an antibody targeting particular cytokine.

For the TNBS experiment, group sizes were as follows: *L. lactis* ILP317, *n* = 10; *L. lactis* REX115, *n* = 10; gavage *L. lactis* ILP317, *n* = 9; and gavage *L. lactis* REX115, *n* = 9.

Statistical significance evaluated by Kruskal-Wallis one-way ANOVA model followed by Dunn´s multiple comparisons test is shown relative to No therapy group of mice. Data are presented as mean ± SD.

**Figure S9. Gating strategy and individual populations of immune cells in the large intestine of mice with experimental DSS-induces colitis.**

**a)** Gating strategy for individual populations: live cells (FVD-eFluor506 negative) were selected, doublets were eliminated (FCS-A vs. FCS-H). Single cells were further analysed based on CD3, CD19, CD11b and CD90 positivity and further gated into all analysed populations (CD4^+^ T cells, intraepithelial T cells, B cells, ILC2, ILC3, eosinophils, macrophages, monocytes and neutrophils). **b)** Analysis of individual cells populations (CD4^+^ T cells, intraepithelial T cells, B cells, ILC2, ILC3, eosinophils, macrophages, monocytes and neutrophils) in DSS induced colitis w/wo all ILP317 and REX115 treatments. **c)** Analysis of individual cells populations (CD4^+^ T cells, intraepithelial T cells, B cells, ILC2, ILC3, eosinophils, macrophages, monocytes and neutrophils) in TNBS induced colitis w/wo all used ILP317 and REX115 treatments. For the DSS experiment, group sizes were as follows: *L. lactis* ILP317, *n* = 10; *L. lactis* REX115, *n* = 10. For the TNBS experiment, group sizes were as follows: *L. lactis* ILP317, *n* = 10; *L. lactis* REX115, *n* = 10; gavage *L. lactis* ILP317, *n* = 9; and gavage *L. lactis* REX115, *n* = 9.

Significant values were calculated by the one-way ANOVA model and Tukey post hoc test. * P *<* 0.05, ** P *<* 0.01, *** P *<* 0.001, **** P *<* 0.0001. Data are presented as mean ± SD.

**Figure S10.** **Experimental replicate – Experimental group 2 for TNBS induced colitis.**

**a)** Scheme of TNBS colitis induction. **b)** DAI for individual mice is expressed as a sum of body weight, stool consistency, and rectal bleeding evaluation. **c)** Colon length; **d)** Inflammatory cell infiltrate; **e)** Mucosal architecture; **f)** Epithelial changes; **g)** Necrosis were evaluated on H&E stained tissue sections from specimens of the last third of colon according to the classification published by Erben *et al*. 2014 (31). **h)** qRT-PCR analysis of IL-1β mRNA transcription in colonic tissue. i) Serum levels of IL-1β, IL-6, IL-22, and TNF-α were measured using fluorescent microbeads analyzed by flow cytometry. All values are displayed for individual mice and as a mean value ± SD for each experimental group. Statistical differences were analyzed by Kruskal-Wallis one-way ANOVA model followed by Dunn´s multiple comparisons test. Statistical significance is shown relative to the group of mice exposed to DSS alone. * P<0.05, ** P<0.01, *** P<0.001. Data are presented as mean ± SD.
